# Structural Probing of Off-Target G Protein-Coupled Receptor Activities within a Series of Adenosine/Adenine Congeners

**DOI:** 10.1371/journal.pone.0097858

**Published:** 2014-05-23

**Authors:** Silvia Paoletta, Dilip K. Tosh, Daniela Salvemini, Kenneth A. Jacobson

**Affiliations:** 1 Molecular Recognition Section, Laboratory of Bioorganic Chemistry, National Institute of Diabetes and Digestive and Kidney Diseases, National Institutes of Health, Bethesda, Maryland, United States of America; 2 Department of Pharmacological and Physiological Science, Saint Louis University School of Medicine, Saint Louis, Missouri, United States of America; Cleveland Clinic Lerner Research Institute, United States of America

## Abstract

We studied patterns of off-target receptor interactions, mostly at G protein-coupled receptors (GPCRs) in the µM range, of nucleoside derivatives that are highly engineered for nM interaction with adenosine receptors (ARs). Because of the considerable interest of using AR ligands for treating diseases of the CNS, we used the Psychoactive Drug Screening Program (PDSP) for probing promiscuity of these adenosine/adenine congeners at 41 diverse receptors, channels and a transporter. The step-wise truncation of rigidified, trisubstituted (at N^6^, C2, and 5′ positions) nucleosides revealed unanticipated interactions mainly with biogenic amine receptors, such as adrenergic receptors and serotonergic receptors, with affinities as high as 61 nM. The unmasking of consistent sets of structure activity relationship (SAR) at novel sites suggested similarities between receptor families in molecular recognition. Extensive molecular modeling of the GPCRs affected suggested binding modes of the ligands that supported the patterns of SAR at individual receptors. In some cases, the ligand docking mode closely resembled AR binding and in other cases the ligand assumed different orientations. The recognition patterns for different GPCRs were clustered according to which substituent groups were tolerated and explained in light of the complementarity with the receptor binding site. Thus, some likely off-target interactions, a concern for secondary drug effects, can be predicted for analogues of this set of substructures, aiding the design of additional structural analogues that either eliminate or accentuate certain off-target activities. Moreover, similar analyses could be performed for unrelated structural families for other GPCRs.

## Introduction

The potential liabilities and advantages of off-target effects of known drugs have been a growing concern in drug development [1]. Often it is difficult to gauge the combined effects of more than one drug action in complex *in vivo* systems, and off-target activities are more commonly viewed as detrimental in the drug discovery process. Therefore, there is interest in understanding the factors affecting drug promiscuity in order to avoid those liabilities early in the drug discovery process. Peters et al. have recently analyzed large datasets of drug-like compounds to identify molecular properties and structural motifs characterizing promiscuous compounds [2]. Keiser et al. have found by *in vitro* screening and prediction new molecular targets of >3600 approved and investigational drugs based on chemical similarity [3]. In some cases, a given off-target activity could be beneficial if it contributes to the net biological effect of the agent in a positive manner [4]. Moreover, off-target effects can also serve as leads for repurposing of known biologically active scaffolds at new molecular targets. This approach was carried out in the past empirically (for example, using privileged scaffolds such as 1,4-dihydropyridines [5]) and can now be performed in a more systematic way with detailed knowledge of the 3D structures of many drug targets including G protein-coupled receptors (GPCRs) [6].

In the course of developing the structure-activity relationship (SAR) of adenosine and adenine derivatives as ligands of nanomolar affinity at the adenosine receptors (ARs) [7], possible off-target binding activities at other GPCRs became evident at higher concentrations than their K_i_ values at ARs [8]. For example, a potent agonist of the A_3_AR, *N* ^6^-(3-iodobenzyl)-5′-*N*-methylcarboxamidoadenosine (IB-MECA), which is now in clinical trials for treating inflammatory diseases [9], was reported in 1994 to interact with serotonin 5HT_2_ receptors, sigma (σ) receptors and peripheral cholecystokinin receptors (binding inhibition of 50–70% at 10 µM) in a broad screen of receptors [8]. Although these unexpected activities typically appeared in the micromolar concentration range, we wondered if drug promiscuity would cause undesirable biological activities and if it was possible to systematically categorize and predict these interactions using receptor 3D modeling. Now with increased interest in the use of AR agonists and antagonists as therapeutic agents, including adenosine and adenine derivatives in addition to ligands of novel chemotypes [9], [10], it was appropriate to re-examine the possible cross-reactivity of AR ligands with diverse receptors and other drug target molecules and try to understand structurally the patterns that emerged. In the case of GPCRs, it seemed possible to understand these off-target interactions according to structural complementarity of the small molecule ligands and their target proteins. With the recent elucidation of the X-ray crystallographic structures of dozens of GPCR-ligand complexes and a large body of mutagenesis data for receptors that have not yet been crystallized [6], [11], it is now feasible to analyze the basis of off-target interactions within the receptor binding sites by modeling and ligand docking.

Drugs that are used for treating disorders of the CNS are especially subject to multiple mechanisms of action, and such polypharmacology can be either advantageous or detrimental [4]. For example, atypical antipsychotic drugs are well served by a finely tuned spectrum of actions at both GPCRs and neurotransmitter uptake sites. It was recognized that many psychoactive drugs have multiple actions, and the effects of each contribution to the overall action of the drugs were not well understood. Efforts have been made to correlate drug promiscuity with chemical and structural characteristics, for example by modifying molecular subdomains while preserving the overall molecular scaffold in matched pairs [12].

The Psychoactive Drug Screening Program (PDSP) at the University of North Carolina, under the direction of Bryan Roth provides a means of testing a multiplicity of receptor interactions of drugs that have CNS effects [13]. Since both agonists and antagonists of the ARs have distinct actions on the CNS [14], and such agents are being considered for the treatment of such conditions as pain, stroke, epilepsy, Parkinson's disease and other neurodegenerative diseases, we generated an array of 10 closely related adenosine/adenine derivatives for examination by the PDSP. The starting structures (5 out of 10) displayed potent (nM) and selective agonist activity at the A_3_AR (Table S1), which is involved in inflammation and cancer and is an experimental approach for the control of chronic neuropathic pain [15]. Thus, it is essential in the preclinical comparison of candidate molecules to analyze promiscuity of interaction of this class of compounds with other targets. The results of the broad screening allowed us to associate structures and substructures with specific interactions with other GPCRs (mainly biogenic amine receptors), ion channels and a transporter. The resulting patterns of SAR were grouped according to similar sets of interactions, as analyzed using molecular modeling. We propose that this analysis will help predict likely off-target effects of other members of the same chemical class. Moreover, this approach can serve as an example for analysis of clusters of structural congeners for other target receptors.

## Results

### Pharmacological Screening

**Figure 1:**
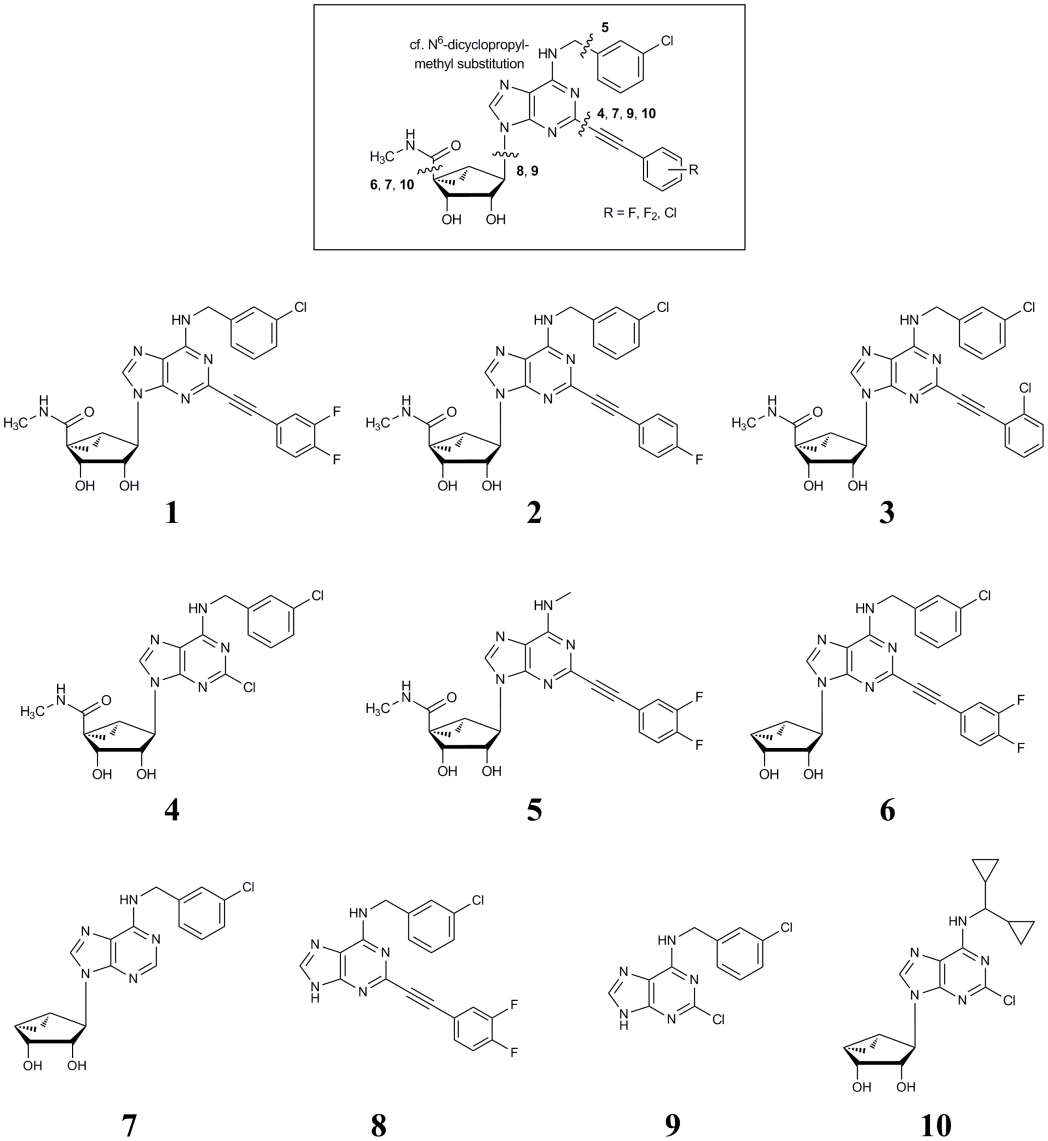
Points of truncation to generate 10 adenosine/adenine derivatives. When present, the ribose-like moiety contains a [3.1.0]bicyclohexane ((N)-methanocarba) ring system designed to maintain an A_3_ and A_1_ ARs preferred conformation, and other substituents are associated with potent activity at these receptors. Using these truncation points, a family of 10 congeners to be evaluated at off-target (non-AR) sites was generated. In one case (compound **10**) an alternate substitution at the N^6^ position was included.

We studied the off-target activities of some of our previously developed AR ligands. In particular, we selected a set of adenosine derivatives that bear a [3.1.0]bicyclohexane ((N)-methanocarba) ring system in place of the tetrahydrofuryl group of ribose in order to reduce conformational flexibility (Figure 1) [16]. This was desired to restrict the range of conformations possible, which would aid in conformational analysis and in docking to protein targets. This ring system constitutes a pseudo-ribose equivalent that is associated with enhanced affinity at the A_3_ and A_1_ AR subtypes [16]. Thus, compounds **1–5** (Figure 1) are potent (nM) agonists of the A_3_AR that lack freedom of twisting of the ribose ring as is present in nucleoside derivatives such as IB-MECA. Compound **10** is an analogue bearing a *N* ^6^-dicyclopropylmethyl substituent, which produces agonist selectivity for the human (h) A_1_AR (K_i_ 49 nM), which is involved in the mechanism of adenosine's antiseizure activity [17].

We removed functionality of this structural series of adenosines/adenine in layers, i.e., by truncating specific groups (Figure 1). Compounds **1–3** contain the full substitution of N^6^, C2, and 5′ positions that are desirable for high A_3_AR affinity across species and full and selective activation of the A_3_AR. Compounds **4** and **5** are truncated at the C2 and *N* ^6^-methyl positions, respectively. Compound **6** (K_i_ at hA_3_AR 100 nM) is truncated at the 4′ position; thus, the A_3_AR potency- and efficacy-enhancing 5′ substituent is absent [18]. Compounds **7–9** contain multiple deletions of the original series, such that in **9** (K_i_ at hA_3_AR 165 nM) only the N^6^ substituted adenine moiety remains. Nucleoside **7** and adenine derivative **8**, with K_i_ at hA_3_AR of 4.9 and 120 nM, respectively, contain either a 4′-truncated (N)-methanocarba ring or an extended C2 substituent (substituted phenylethynyl). In general, a greater degree of truncation was associated with a diminished ability to activate ARs, although receptor binding may be maintained. Thus, potent AR agonists were converted into AR antagonists, as discussed elsewhere [19], [20]. The binding affinity of compounds **1–10** at three subtypes of ARs is given in Table S1.

Because of the considerable interest in using AR ligands for treating diseases of the CNS [14], we used the services of the PDSP for screening this family of ligands at 41 binding sites that include other GPCRs, ion channels, and transporters (complete list reported in Text S1). As is standard for the PDSP, an initial screen was performed at 10 µM of each compound, generally by radioligand binding but in some cases using functional assays. Those compounds that inhibited the specific binding or induced the effect by >50% of maximal (in at least one experiment) were measured in full concentration-response curves. The results for all of the molecular targets with a measured K_i_ <10 µM for at least one of the 10 compounds are given in Table 1. Complete results of the primary screening for all the tested receptor sites are shown in Table S2 and representative full curves for each compound at off-target sites are reported in Figure S1.

**Table 1:** Potency of a series of (N)-methanocarba adenosine and adenine derivatives (AR ligands) at off-target GPCRs, ion channels and a transporter.

		Binding assays, unless noted. K_i_ (µM) or *% inhibition* at 10 µM^a^									
Target	Family	1	2	3	4	5	6	7	8	9	10
**GPCRs**											
**α_2A_**	adrenergic	**4.77±1.43**	*6%*	*22%*	*26%*	*0%*	*14%*	*26%*	**2.19±0.27**	**3.00±0.51**	*17%*
**α_2B_**	adrenergic	**2.86±1.11**	*12%*	**6.48±2.94**	*21%*	*0%*	**5.97±1.53**	**6.34±1.47**	**1.09±0.14**	**0.061±0.02**	*15%*
**α_2C_**	adrenergic	**2.04±0.95**	**3.64±1.37**	**1.81±0.25**	*11%*	*13%*	**5.40±1.22**	**5.36±1.25**	**1.02±0.69**	**0.314±0.071**	*0%*
**β_3_**	adrenergic	**1.45±0.51**	**1.56±0.56**	**1.17±0.19**	*0%*	**2.32±0.39**	**2.46±0.13**	*4%*	*5%*	*0%*	*0%*
**H_4_**	histaminergic	*17%*	*28%* c	*9%* c	ND	ND^c^	ND	*0%*	*5%*	*5%*	*6%*
**5HT_1A_**	serotonergic	**7.62** b	*5%*	*6%*	*29%*	*10%*	*9%*	*28%*	*11%*	*12%*	*4%*
**5HT_2B_**	serotonergic	**2.58±0.22**	**2.13±0.27**	**3.65±1.05**	**0.075±0.007** d	*4%*	*34%*	**2.22±0.65**	**4.00±1.92**	**1.78±0.35** e	**0.641±0.243**
**5HT_2C_**	serotonergic	**7.19±1.41**	*16%*	*25%*	**0.122±0.017** d	*1%*	*0%*	**1.74** (1)	*11%*	**3.32±0.32** e	**1.85±0.46**
**5HT_5A_**	serotonergic	*3%*	*25%*	**6.30** b	*21%*	*5%*	*15%*	*16%*	*11%*	**4.49** b	*0%*
**5HT_7_**	serotonergic	*5%*	*6%*	*8%*	*40±7* **7.73** (1)	*0%*	*0%*	*35±2* **3.92** (1)	*0%*	*12%*	*0%*
**δ**	opioid	**2.44±1.54**	**6.62±1.70**	*31%*	*10%*	*9%*	*11%*	*7%*	*12%*	*7%*	*0%*
**Ion channels**											
**5HT_3_**	serotonergic	*3%*	*26%*	*50%*	*5%*	*2%*	*0%*	**3.26±0.80**	*0%*	*28%*	*3%*
**hERG** c	potassium	**12.2**	**39.5**	**39.2**	*2%*	**7.93**	ND	ND	ND	ND	*0%*
**Other receptors**											
**σ_1_**	Dimethyl-tryptamine^f^	*0%*	*2%*	*33%*	*2%*	*10%*	*16%*	*29%*	**1.68** (1)	*0%*	*9%*
**σ_2_**	unknown	**0.908±0.294** (2)	*30%*	*42%*	*0%*	*33%*	**2.94±1.50** (2)	**2.68±0.47** (2)	*14%*	*0%*	*0%*
**Transporter**											
**PBR**	peripheral benzodiazepine	**0.340±0.072** (2)	**0.253±0.057** (2)	**0.344±0.149** (2)	*32%*	*25%*	**1.75±0.36** (2)	*13%*	*13%*	*8%*	*0%*

a All experiments were binding assays, unless noted, performed by the PDSP. % values were from single concentration (10 µM) determination. A value determined as <*0%* is represented as *0%* here (within experimental error). Ki values were determined from full concentration response curves only for receptors that displayed >*50%* inhibition at 10 µM for at least one of the listed compounds. n = 3-6, unless noted in parentheses. K_i_ values <10 µM are shown in bold. Other receptors tested for binding in single concentration determination are: 5HT_1B_, 5HT_1D_, 5HT_1E_, 5HT_2A_, 5HT_6_, α_1A_, α_1B_, α_1D_, β_1_, β_2_, D_1_, D_2_, D_3_, D_4_, D_5_, GABA_A_, H_2_, H_3_, M_1_, M_2_, M_3_, M_4_, M_5_, κOR, µOR.

b Based on data with one or two full inhibition curves that provided K_i_ values <10 µM; other curves did not reach 50% inhibition at the max. concentration tested (10 µM) and extrapolated values were averaged.

c Functional assays were performed: hERG assay (shown in table); H_4_ Tango™ antagonist assay: Compounds 2, 3 and 5 at 10 µM inhibited activity by 28±7%, 58±16% and 55±11%, respectively, and were inactive in a H4 Tango™ agonist assay.

d **4**, at 10 µM in functional assays was nearly inactive as 5HT_2B_ agonist (*4.0%* of full agonist) and 5HT_2C_ agonist (*4.6%* of full agonist); antagonism of **4** was measured by inhibition of agonist activity at 5HT_2B_ (IC_50_ 887 nM) and 5HT_2C_ (IC_50_ 3.26±0.80 µM).

e **9**, at 10 µM in functional assays was nearly inactive as 5HT_2B_ agonist (*4.4%* of full agonist) and 5HT_2C_ agonist (5.*9%* of full agonist); but active as antagonist at 5HT_2B_ (*26.4%* inhibition) and 5HT_2C_ (*65.1%* inhibition).

f One of the putative endogenous ligands.

ND, not determined.

Several biogenic amine receptors, such as α-adrenergic and serotonin (5HT) receptors, were revealed as interaction sites. The most potent interactions were found for a 5′-*N*-methyluronamide **4** at 5HT_2B_ serotonergic receptors (K_i_ 75 nM) and 4′-truncated compound **9** at α_2B_ adrenergic receptors (K_i_ 61 nM). Other potent interactions (K_i_ <1 µM) at off-target GPCRs were seen for the following: adenine derivative **9** at α_2C_ receptors (K_i_ 0.31 µM); compound **4** at 5HT_2C_ receptors (K_i_ 0.12 µM); compound **10** at 5HT_2B_ receptors (K_i_ 0.64 µM). Moreover, binding in the low µM range (K_i_ <5 µM) was found for some compounds at several GPCRs such as 5HT_2B_ and 5HT_2C_ serotonergic receptors; α_2A_, α_2B_ and α_2C_ adrenergic receptors; β_3_ adrenergic receptor and δ opioid receptor. Therefore, we performed docking studies of the appropriate adenosine congeners at those GPCRs showing K_i_ values in the low µM range for several compounds. The 5HT_7_ serotonergic receptor was also included in this analysis, because there was a variable degree of radioligand inhibition, with some values close to 50% at 10 µM.

Pharmacological screening of the known A_3_AR agonist IB-MECA detected binding at 5HT_2B_ and 5HT_2C_ serotonergic receptors, with K_i_ values of 1.08 µM and 5.42 µM respectively, and no other off-target interactions.

At non-GPCRs, fully substituted nucleosides **1–3** bound tightly at the peripheral benzodiazepine receptor (PBR, a transporter, K_i_ 0.2–0.3 µM). 4′-Truncated nucleoside **6** bound less potently (K_i_ 1.7 µM) at the PBR. Derivative **7** inhibited binding at 5HT_3_ ion channels (K_i_ 3.26 µM). Binding in the low µM range was found for adenine derivative **8** at the σ_1_ receptor and for compounds **1**, **6** and **7** at the σ_2_ receptor.

Functional assays of compounds **4** and **9** at 5HT_2B_ and 5HT_2C_ receptors indicated lack of agonist action (Figure S2), although the antagonism was not always complete at 10 µM (at 5HT_2B_ and 5HT_2C_ receptors, respectively, 60% and 94% inhibition by **4**; 26% and 65% inhibition by **9**). Moreover, compound **9** was found to be an antagonist with an IC_50_ of 2.9 µM in a functional assay at the α_2C_ adrenergic receptor. Several compounds were also tested in a Tango™ functional assay (Invitrogen, Life Technologies) of the H4 histamine receptor. Only 5′-N-methyluronamides 3 and 5 showed significant antagonist potency at 10 µM (inhibition of 58±16% and 55±11%, respectively, n = 4). Some compounds were also tested in a functional assay at the hERG potassium channel, and the inhibition was either absent or in the >7 µM range.

**Figure 2:**
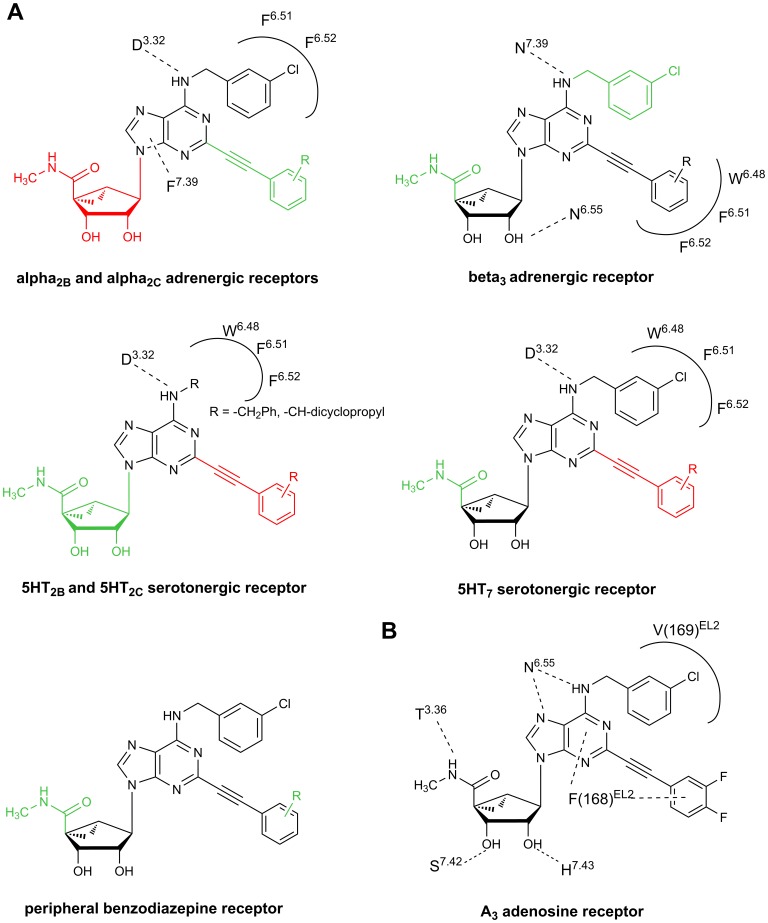
Definition of pharmacophore structures for individual off-target receptor sites. (A) Colors code the degree of tolerance of appended groups: pharmacophores (minimum structural requirement for binding, shown on **1** as template) are shown in black, favorable or tolerated substituents are shown in green and not tolerated substituents are shown in red. Some residues predicted to be in contact with the adenosine derivatives at the off-target receptors are highlighted according to the explanations provided in the text (corresponding to poses shown in Figure 4B for the h5HT_2_ receptors and Figure 7B for the hβ_3_ receptor. This is an approximation based on a limited set of compounds. Pharmacophores for other targets were not well defined with the current data set, and weak hits correspond to individual compounds as noted in Table 1. (B) A comparison with the residues in contact with compound **1** at the A_3_AR, as previously predicted by docking studies [16].

By examining the off-target (i.e., non-AR) interactions within this closely related series of congeners, for some receptors it was possible to correlate the appearance of a given interaction and its structural requirements in a systematic manner. Figure 2A shows a summary of the pharmacophores associated with binding activity at the various off-target GPCRs. It must be noted that this is an approximation based on a limited set of compounds and will require examination of additional analogues to provide a more precise definition. The recognition patterns for different GPCRs were clustered according to which substituent groups were tolerated. Adrenergic receptors α_2B_ and α_2C_ cluster together with the characteristic that the best affinity is shown for compound **9** and the extended C2 substituent does not enhance the affinity but can be tolerated, while the pseudosugar moiety (bicyclic ring system) is more detrimental. At the β_3_ adrenergic receptor the presence of both the C2-phenylethynyl group and the pseudosugar ring is required for binding. However, the *N* ^6^-(3-chlorobenzyl) group and the 5′-methyluronamide are tolerated but not required. At 5HT_2B_ serotonergic receptors different substitutions at the *N* ^6^ position are tolerated, and a 5′-*N*-methyluronamide group is a favorable factor. At 5HT_2C_ serotonergic receptors similar requirements for binding were observed, but fewer deviations from the structure of derivative **4** are tolerated. At the 5HT_7_ receptor the presence of the *N* ^6^-(3-chlorobenzyl) group and the pseudosugar ring is required, while the presence of a C2-phenylethynyl substituent abolished binding. Binding at the PBR is associated with the concomitant presence of the methanocarba ring and both C2 and *N* ^6^ substituents; the 5′-*N*-methyluronamide group is tolerated but not required.

### Molecular modeling

We performed molecular docking studies to rationalize the binding data of the adenosine congeners at several members of the GPCR family, trying to understand the basis for particular structural requirements. In particular, we focused on those receptors that bound at least one compound with a K_i_ lower than 1 µM (α_2B_ and α_2C_ adrenergic receptors, 5HT_2B_ and 5HT_2C_ serotonergic receptors) or that showed a recognition pattern within our series of compounds (β_3_ adrenergic receptor and 5HT_7_ serotonergic receptor). For the receptors of interest, we used the crystallographic structural information when available or we built homology models based on close crystallographic templates. Sequence alignments used to build homology models and boundaries of the boxes used for docking studies are reported in Figures S3 and S4, respectively. To validate the docking and homology modeling approaches we performed self-docking of co-crystallized ligands at the receptor X-ray structures used in the study and docking of known ligands at target receptors. Crystallographic poses of the crystals used in the present study and results of self-docking are reported in Figure S5 for comparison with the proposed binding modes of the adenosine congeners. Results of self-docking showed that the top ranking pose obtained for a given ligand in the docking protocol reproduced the crystallographic structure of the complexes, as illustrated in the superposition of the docking poses with the crystallographic data (Figure S5). Docking poses obtained for other known aminergic ligands at selected target receptors are reported in Figure S6. In general, both crystals and models used in this study showed reasonable docking poses for several known ligands. Binding modes at various aminergic receptors were similar, with the charged amino group of the ligands located in proximity to the highly conserved aspartic acid in transmembrane helix (TM) 3 and a hydrophobic group occupying the lower part of the binding site delimited by conserved aromatic residues in TM6 and TM7. Smaller ligands occupied only the lower part of the cavity, while larger compounds additionally interacted with residues in the upper part of the TMs and extracellular region in different ways depending on their steric and chemical features. Docking results were consistent with reported crystallographic complexes of the target receptors with different compounds, if available, or of other receptors of the same subfamily.

#### α adrenergic receptors

To date, no crystallographic data have been published for the α adrenergic receptor family. Among the GPCRs whose structures have been solved, the hD_3_ dopaminergic receptor showed the highest identity percentage with both α_2B_ and α_2C_ adrenergic receptors (≈30%), followed by the h5HT_1B_ serotonergic receptor (≈28%) and the turkey β_1_ adrenergic receptor (≈28%).

Docking of the adenosine congeners at homology models of the hα_2B_ and α_2C_ adrenergic receptors based on the hD_3_ dopaminergic receptor crystal structure (PDB ID: 3PBL) [21] did not give reasonable results. In fact, the lower part of the binding site was too tight to accommodate the ligands and especially the bulkier derivatives. Therefore, we also built models of the α_2B_ and α_2C_ adrenergic receptors based on a h5HT_1B_ serotonergic receptor structure (PDB ID: 4IAR) [22] and a turkey β_1_ adrenergic receptor structure (PDB ID: 4AMJ) [23]. Better docking results in terms of a binding site fit were obtained at the 5HT_1B_ receptor-based models. In fact, at the β_1_ receptor-based models the binding site was shallow, and the conserved Asp in TM3 (residue 3.32 using the Ballesteros-Weinstein notation) [24] was not accessible. Differences observed in ligand docking to hα_2B_ and hα_2C_ receptor models based on different templates seem not to be related to the agonist- or antagonist-bound state of the template but more likely to the different overall arrangement of the helices in the template receptors; in fact, also docking of know adrenergic ligands, both agonist and antagonist, did not give good results at the D_3_-based and β_1_-based α_2_ adrenergic receptor models. This made the h5HT_1B_ receptor more suitable for building a model of the hα2 receptor family compared to the other templates tested. Therefore, we investigated in depth the binding modes of the adenosine congeners at the 5HT_1B_ receptor-based models.

**Figure 3:**
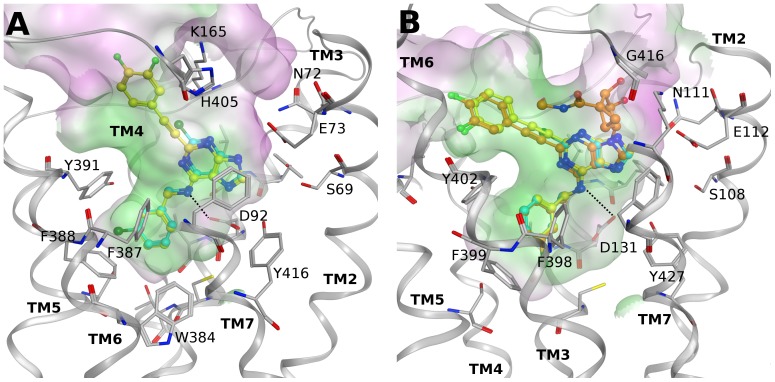
Docking at α adrenergic receptors. Hypothetical binding modes of selected compounds at homology models of the hα_2B_ and hα_2C_ adrenergic receptors based on the h5HT_1B_ receptor structure. (A) Compounds **8** (yellow carbons) and **9** (cyan carbons) at the α_2B_ receptor. (B) Compounds **1** (orange carbons), **8** (yellow carbons) and **9** (cyan carbons) at the α_2C_ receptor. Ligands are show in ball and stick and some residues important for ligand recognition are shown in stick (gray carbons). Hydrogen atoms are not displayed. H-bonds are shown as black dashed lines. The Connolly surface of the amino acids surrounding the binding site is displayed. Surface color indicates the lipophilic potential: lipophilic regions (green), neutral regions (white) and hydrophilic regions (magenta).

Figure 3A shows hypothetical binding poses of adenine derivatives **8** and **9** at the α_2B_ adrenergic receptor obtained after docking studies. According to this binding mode, these compounds orient the *N* ^6^-(3-chlorobenzyl) group toward the lower part of the binding site in a hydrophobic pocket delimited by Val93 (3.33), Trp384 (6.48), Phe387 (6.51), Phe388 (6.52) and Tyr391 (6.55). The adenine core forms aromatic interactions with Phe412 (7.39), and the exocyclic amino group interacts through a H-bond with the conserved Asp in TM3, i.e., Asp92 (3.32). In the binding pose of **8** the C2-phenylethynyl group is directed toward the extracellular region in proximity to TMs 5 and 6. In fact, the binding site opening to the extracellular side is wider in proximity to these helices than it is on the cytosolic side near TMs 2 and 7.

A similar binding mode to that observed at the α_2B_ adrenergic receptor was found for compounds **8** and **9** at the α_2C_ receptor subtype, as shown in Figure 3B. The main interactions formed by the nucleobase and the *N* ^6^ substituent are conserved, and the C2-phenylethynyl group of **8** is directed toward TMs 5 and 6. However, in this case the extracellular region near TMs 2 and 7 is slightly wider and could more easily accommodate larger compounds, such as the ones bearing the pseudosugar ring (see the pose of fully substituted nucleoside **1** in Figure 3B), as compared to the α_2B_ subtype. In fact, a comparison between the α_2B_ and α_2C_ adrenergic receptor models showed very high conservation of the lower part of the binding sites between the two subtypes, while the main differences are located in the second and third extracellular loops (EL2 and EL3) and in the upper part of TMs 6 and 7. In particular, two bulky residues whose side chains are inclined on top of the binding site at the α_2B_ receptor, i.e., Lys165 in EL2 and His405 (7.32) in TM7, are reduced in size as Gly residues at the α_2C_ adrenergic receptor (Gly203 and Gly416, respectively), which allows the pseudo-sugar to bind better.

#### Serotonergic (5HT) receptors

The crystal structure of the h5HT_2B_ serotonergic receptor in complex with ergotamine (PDB ID: 4IB4) [25] was used to study the binding modes of our derivatives at this subtype and also as template to build a homology model of the h5HT_2C_ serotonergic receptor. However, a homology model of the h5HT_7_ serotonergic receptor was based on the h5HT_1B_ receptor crystal structure (PDB ID: 4IAR) [22], because of their slightly higher sequence identity.

**Figure 4:**
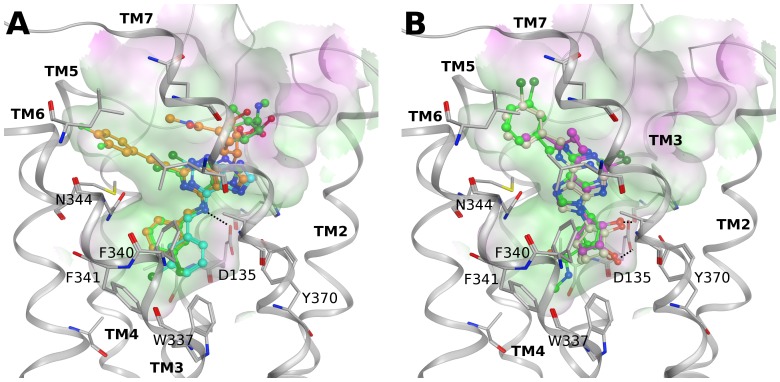
Docking at 5HT_2B_ serotonergic receptor. Hypothetical alternative binding modes of selected compounds at the h5HT_2B_ receptor crystal structure. (A) First proposed binding mode for compounds **4** (green carbons), **7** (pale pink carbons) and **10** (magenta carbons) at the 5HT_2B_ receptor. (B) Second proposed binding mode for compounds **1** (orange carbons), **4** (green carbons) and **9** (cyan carbons) at the 5HT_2B_ receptor. Ligands are shown in ball and stick and some residues important for ligand recognition are shown in stick (gray carbons). Hydrogen atoms are not displayed. H-bonds are shown as black dashed lines. The Connolly surface of the amino acids surrounding the binding site is displayed. Surface color indicates the lipophilic potential: lipophilic regions (green), neutral regions (white) and hydrophilic regions (magenta).

At the 5HT_2B_ serotonergic receptor, derivatives not bearing an extended C2 substituent (compounds **4**, **7** and **10**) showed two main possible binding modes (Figure 4). In the first proposed binding pose (Figure 4A and PDB File S1) the methanocarba moiety is located in the lower part of the binding site interacting with Val136 (3.33), Ser139 (3.36), Thr140 (3.37), Phe340 (6.51), Phe341 (6.52), Val366 (7.39) and Tyr370 (7.43); moreover the two hydroxyl groups form H-bonds with the conserved Asp135 (3.32). The *N* ^6^ substituent is oriented towards the extracellular region comprised of EL2, TM6 and TM7. On the other hand, the second binding mode (Figure 4B and PDB File S2) presents the *N* ^6^-(3-chlorobenzyl) group pointing towards the intracellular side of the cavity with the exocyclic NH forming a hydrogen bond with Asp135 (3.32) and the phenyl ring making hydrophobic contacts with Val136 (3.33), Ser139 (3.36), Trp337 (6.48), Phe340 (6.51), Phe341 (6.52) and Tyr370 (7.43). The adenine core is stabilized by interactions with Met218 (5.39), Phe340 (6.51), Asn344 (6.55) and Val366 (7.39). The pseudosugar moiety interacts mainly with residues of EL2 and with Glu363 (7.36). This orientation also allows C2-phenylethynyl derivatives to fit the cavity and adenine derivatives lacking a pseudosugar ring to bind, as depicted by the poses of fully substituted nucleoside **1** and adenine derivative **9** in Figure 4B, and therefore can explain their affinity for this subtype.

At the 5HT_2C_ serotonergic receptor, the adenosine congeners docked in similar fashion as with the 5HT_2B_ subtype (data not shown) in agreement with the similar binding pattern of this series at these two subtypes. The main residues making ligand contact that are located in the lower part of the binding site are conserved between the two receptors, while some differences are observed in the upper TM region and in the ELs in proximity with the docked compounds. In particular, the extracellular end of TM5 and the C-terminal part of EL2 present several different residues and also a different length (there are 3 more residues at the 5HT_2B_ subtype). Therefore, the alignment to build the 5HT_2C_ model cannot be very accurate in this area that is likely to be a region determining selectivity among different serotonergic subtypes.

**Figure 5:**
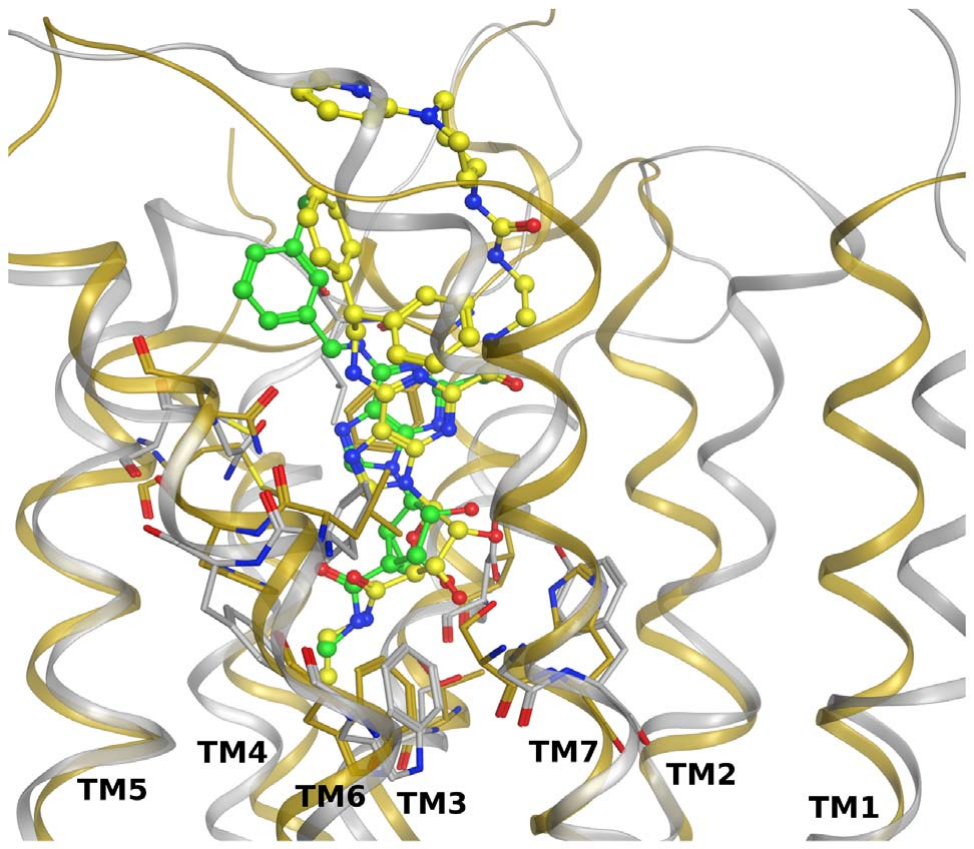
Similarity of binding between 5HT_2B_ serotonergic receptor and adenosine receptors. Comparison between the docking pose of compound **4** (green carbons) at the 5HT_2B_ serotonergic receptor structure (silver ribbon) as shown in Figure 4A and the crystallographic pose of the AR agonist UK-432097 (yellow carbons) at the hA_2A_AR (gold ribbon). Ligands are shown in ball and stick, and some residues important for ligand recognition are shown in stick (silver or gold carbons). Hydrogen atoms are not displayed.

It is interesting to note that there is a high similarity between the first proposed binding pose of compounds bearing small substituents at the C2 position (compounds **4**, **7** and **10**) at these serotonergic receptors and their binding mode at ARs (as shown by previous docking studies and crystallographic poses of analog compounds). In fact, they present a similar orientation in the binding sites of the two class of receptors as shown by the superposition of the docking pose of compound **4** at the 5HT_2B_ receptor and the crystal pose of the nucleoside derivative UK-432097 at the hA_2A_AR (PDB ID: 3QAK) [26] in Figure 5. Residues in contact with the ligands in the two different receptors belong to similar positions in the TM region, but the amino acid types are very different.

**Figure 6:**
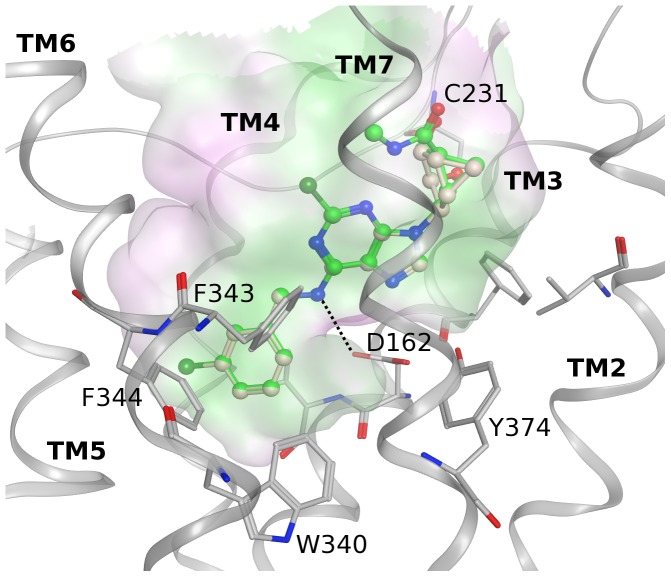
Docking at 5HT_7_ serotonergic receptor. Hypothetical binding mode of compounds **4** (green carbons) and **7** (pale pink carbons) at a homology model of the h5HT_7_ serotonergic receptor based on the h5HT_1B_ receptor structure. Ligands are shown in ball and stick, and some residues important for ligand recognition are shown in stick (gray carbons). Hydrogen atoms are not displayed. H-bonds are shown as black dashed lines. The Connolly surface of the amino acids surrounding the binding site is displayed. Surface color indicates the lipophilic potential: lipophilic regions (green), neutral regions (white) and hydrophilic regions (magenta).

At the 5HT_7_ serotonergic receptor, only compounds **4** and **7** showed a significant degree of binding inhibition (40% and 35% at 10 µM, respectively). The binding poses of the two compounds at this receptor showed an orientation in the cavity similar to the second binding mode proposed at the 5HT_2B_ and 5HT_2C_ serotonergic receptors, with the *N* ^6^-(3-chlorobenzyl) group pointing towards the inner side of the binding site and the pseudosugar moiety directed towards the extracellular side (Figure 6). Similar interactions as observed for the other serotonergic receptors are established with this subtype. However, the cavity apppeared to be smaller as compared to the 5HT_2B_ and 5HT_2C_ receptors, and this can be an indication of the null affinity of bulkier compounds at this subtype.

#### Other GPCRs

**Figure 7:**
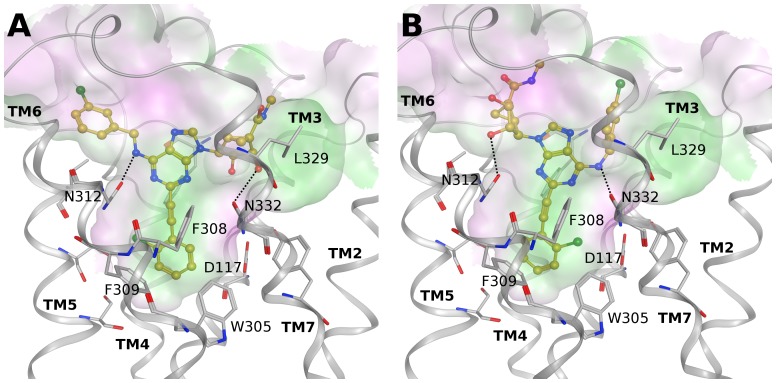
Docking at β_3_ adrenergic receptor. Hypothetical alternative binding modes of compound **3** (yellow carbons) at a homology model of the hβ_3_ adrenergic receptor based on the turkey β_1_ adrenergic receptor structure. In both cases (A and B), the C2-arylethynyl group is deeply buried in the binding site. Ligands are shown in ball and stick and some residues important for ligand recognition are shown in stick (gray carbons). Hydrogen atoms are not displayed. H-bonds are shown as black dashed lines. The Connolly surface of the amino acids surrounding the binding site is displayed. Surface color indicates the lipophilic potential: lipophilic regions (green), neutral regions (white) and hydrophilic regions (magenta).

Among the other GPCRs that bound some of the adenosine congeners in the low µM range (α_2A_ adrenergic receptor, β_3_ adrenergic receptor and δ opioid receptor), the β_3_ adrenergic receptor showed the highest hit rate. To date, several crystallographic structures of the turkey β_1_ and hβ_2_ adrenergic receptors are available; however, there are no published structures of the third member of this receptor class. Therefore, we built a homology model of the hβ_3_ adrenergic receptor based on the turkey β_1_ adrenergic receptor crystal structure (PDB ID: 4AMJ) [23] that showed a slightly higher percentage of sequence identity (≈48%). Figure 7 shows two hypothetical binding modes of compound **3** at this receptor obtained after molecular docking simulations. In both the proposed alternative binding modes, the C2-phenylethynyl group is located in the lower part of the binding cavity surrounded by Asp117 (3.32), Val118 (3.33), Val121 (3.36), Ser208 (5.42), Phe213 (5.47), Trp305 (6.48), Phe308 (6.51) and Phe309 (6.52). The adenine core is interacting with Phe198 in EL2 and Phe328 (7.35) in TM7, while the exocyclic NH or the hydroxyl groups of the methanocarba ring could form H-bonds with either Asn312 (6.55) or Asn332 (7.39).

### Correlation of residues involved in GPCR interactions

Starting from all the previously proposed binding modes we analyzed the residues in contact with the highest affinity ligand at each studied receptor and we compared them with the residues in contact with compound **1** previously docked at the hA_3_AR [16]. Residues within 4 Å from each docked ligand at different receptors are listed in Table 2 and key residues for the interaction with off-target sites and with the hA_3_AR are depicted in Figure 2A and Figure 2B, respectively. It can be noted that for the majority of receptors the residues in contact with the ligands are located in TMs 3, 5, 6 and 7. Moreover, topologically equivalent residues previously shown to make consensus contacts with diverse ligands in nearly all the reported crystallographic structures of family A GPCRs, such as residues at positions 3.32, 3.33, 3.36, 6.48, 6.51 and 7.39, [11] are also in proximity of our docked compounds in the studied receptors. There is a high conservation of the residues at these positions among the biogenic amine receptors explored in this study, with Asp at 3.32, Val at 3.33, Trp at 6.48 and Phe at 6.51. In addition to these residues, another conserved contact among all the analyzed receptors is with residue 6.55. An Asn residue at this position is highly conserved among ARs and is key in anchoring both AR agonists and antagonists. An Asn residue is present at this position also at the β_3_ adrenergic receptor and 5HT_2B_ and 5HT_2C_ serotonergic receptors, and it occurs as Tyr at the α_2B_ and α_2C_ adrenergic receptors.

**Table 2:** Comparison of TM residues located within 4 Å from the docking pose of the most potent compound at each analyzed off-target biogenic amine GPCR.

Residue Number	α_2B_ receptor compound 9	α_2C_ receptor compound 9	β_3_ receptor compound 3	5HT_2B_ receptor compound 4	5HT_2C_ receptor compound 4	A_3_AR compound 1
2.61	Ser 69	Ser108				Ala69
2.64	Asn72	Asn111				Val72
2.65	Glu73	Glu112				
3.28	Tyr88	Tyr127	Trp113	Trp131		
3.29	Leu89	Leu128		Leu132		
3.32	Asp92	Asp131	Asp117	Asp135	Asp134	Leu90
3.33	Val93	Val132	Val118	Val136	Val135	Leu91
3.36	Cys96	Cys135	Val121	Ser139	Ser138	Thr94
3.37	Thr97		Thr122	Thr140	Thr139	His95
3.40				Ile143	Ile142	Ile98
5.35						Met174
5.36					Pro212	
5.38						Met177
5.39			Val205	Met218	Val215	
5.42			Ser208			Ser181
5.46	Ser180	Ser218	Ser212	Ala225	Ala222	
5.47						Ile 186
6.48	Trp384		Trp305	Trp337	Trp324	Trp243
6.51	Phe387	Phe398	Phe308	Phe340	Phe327	Leu246
6.52	Phe388	Phe399	Phe309	Phe341	Phe328	
6.54						Ile249
6.55	Tyr391	Tyr402	Asn312	Asn344	Asn331	Asn250
6.58			Arg315	Leu347		Ile253
6.59				Val348	Val335	
7.32			Gly325	Gln359	Glu347	
7.35			Phe328	Leu362	Leu350	Leu264
7.36		Lys420	Leu329		Asn351	Tyr265
7.39	Phe412	Phe423	Asn332	Val366	Val354	Ile268
7.42						Ser271
7.43	Tyr416	Tyr427		Tyr370	Tyr358	His272

The residues in contact with compound **1** in the hA_3_AR docking pose are reported for comparison. The Ballesteros-Weinstein numbering is reported in the first column.

## Discussion

Polypharmacology at GPCRs can be a liability or an opportunity depending on which receptors and which compounds are involved, and screening of off-target binding interactions during drug discovery and development is important to predict possible secondary drug actions [27]. The pharmacological screening presented in this paper revealed some off-target interactions for a series of adenosine/adenine congener molecules that are highly engineered for interaction with ARs. In general, the off-target profile of the adenosine congeners is in agreement with previous studies on the off-target activities of large datasets of drugs and drug-like compounds. In fact, these analyses [2] show that biogenic amine receptors attract the highest hit rate followed by transporters of biogenic amines, σ receptors and opioid receptors. The target hit rate at aminergic GPCRs increases for positively charged compounds, and even more if these compounds are also lipophilic or have two or more aromatic rings. Even though the adenosine congeners do not have a positive charge at physiological pH, they showed a high hit rate toward the biogenic amine receptors.

Multiple sequence alignments and phylogenetic analyses located the ARs in a branch of the family A GPCRs containing 64 receptors divided into two major clusters [28]. The first MECA (Melanocortin, Endothelial, Cannabinoid, and Adenosine) cluster includes receptors with which the ARs share the most recent common evolutionary origin; the second cluster encompasses all the receptors for biogenic amines. Interestingly, ARs show a high sequence similarity with biogenic amine receptors but are predicted to be more recent in evolution as are other members of the MECA cluster [28]. Furthermore, mutagenesis data proposed a parallelism between ARs and biogenic amine receptors, identifying important common regions for ligand recognition, such as the essential Asp 3.32 of the biogenic amine receptors and the corresponding Val of the A_2A_AR [29]. This highly conserved Asp residue in TM3 of biogenic amine receptors acts as a counterion for the positively charged amino group of the native ligands. Consistent with this proximity on the GPCR dendrogram [28], [30], there was considerable appearance of off-target interactions of our AR ligands at biogenic amine receptors.

To understand why some of the adenosine congeners bound strongly to particular aminergic receptors, we studied their possible binding modes trying to recognize the structural features required for the interaction. In some cases, X-ray structures were available already for modeling recognition at the unanticipated interacting GPCRs, and in other cases homology models were obtained from closely related templates [22], [23], [25].

Several of the adenosine congeners interacted with the three subtypes of the α_2_ adrenergic receptor family, while no significant binding was observed for any of the compounds at the α_1_ adrenergic subtypes. In particular, binding in the sub-µM range was found for adenine derivative **9** at the α_2B_ and α_2C_ adrenergic receptors (K_i_ = 61 nM and K_i_ = 314 nM, respectively). The docking of this compound highlighted a set of minimum interactions required for binding of this truncated pharmacophore at α_2_ adrenergic receptors. The larger adenine derivative **8** could still fit in their binding sites orienting the extended C2 substituent toward TMs 5 and 6. However, the accommodation of sterically bulkier compounds (bearing both the C2-phenylethynyl group and the methanocarba ring) proved to be difficult because there was limited space in the extracellular region in proximity to TMs 2 and 7. It has to be noted that the cavity of the α_2C_ adrenergic receptor was wider as compared to the α_2B_ subtype and could fit larger ligands therefore tolerating, slightly better, the simultaneous presence of an extended C2 substituent and a methanocarba ring. Such proposed binding mode for this series of compounds agrees with the structural requirement for binding at these receptors as shown in Figure 2. Even though the binding mode at the α_2A_ adrenergic receptor was not analyzed in detail in this study, pharmacological results at this subtype showed a binding pattern for the adenosine congeners (i.e. defined binding of **1**, **8** and **9**) similar to that observed at the α_2B_ and α_2C_ receptors, but with lower potency overall. Therefore, a similar binding mode of the adenosine congeners likely occurs even at the α_2A_ receptor, also considering that residues making ligand contact in the TM region are highly conserved among these three adrenergic subtypes, and possibly the degree of potency can be modulated by differences among the residues in the EL region.

The adenosine congeners did not bind the β_1_ and β_2_ adrenergic receptors but low µM K_i_ values were found for some compounds at the β_3_ adrenergic receptor. K_i_ values above the nM range suggest that these ligands could fit the binding site but did not bind very tightly. In fact, the proposed binding modes highlighted a good shape complementarity with the cavity, but no interaction with the conserved Asp in TM3 was observed. Moreover, the C2 substituent was anchored in the lower part of the binding site in a hydrophobic subpocket, in agreement with the fact that only compounds bearing an extended C2 group bound to this receptor. Comparison of the three subtypes of the β adrenergic receptor family (Figure S7, panels A and B) showed a very similar overall arrangement of the TM helices and a very high conservation of residues in contact with the ligand with only a few differences in the residues located in the upper part of the binding cavity. Therefore, the lack of binding of **3** at the β_1_ and β_2_ receptors could be due to differences at the entrance of the binding site that could influence the orientation of the ligand in the cavity or its possible approach process. In particular, several small residues (Ala or Gly) in the β_3_ receptors are mutated to bulkier and sometimes charged residues in the other two subtypes, and this can present a completely different scenario as the ligand approaches the receptor.

Previous screening studies have shown that, in general, among the biogenic amine receptors, serotonergic GPCRs, and in particular the 5HT_2B_ receptor, exhibit very high hit rates for drug-like compounds [2]. In agreement with this observation, some serotonergic receptors were revealed as off-target sites of several of the adenosine congeners, with the 5HT_2B_ receptor binding two compounds in the nM range. Nucleoside **4** was the most potent compound at both the 5HT_2B_ and 5HT_2C_ receptors. After analysis of the docking results, two possible binding modes were proposed for this compound at both receptors. The first docking pose, presenting two H-bonding interactions between the pseudosugar hydroxyl groups and the conserved Asp 3.32, shows an orientation in the binding cleft very similar to that adopted by nucleoside derivatives at the ARs. This docking mode was found at 5HT_2_ receptors only for compounds not bearing an extended C2 substituent. On the other hand, the second orientation can explain the binding of compounds bearing either small or bulky C2 substituents and requires the presence of a bulky N^6^ substituent to fill the lower part of the binding site below Asp 3.32. Moreover, a similar orientation, but without a tolerance for extended C2 groups, has been observed for compounds **4** and **7** at the 5HT_7_ receptor. Considering that this second orientation is common to different receptor subtypes and can rationalize the binding of all the compounds of this series in agreement with their binding requirement, it seems more likely to be a reasonable binding mode for the serotonergic receptor family. Further studies on the off-target activities of other AR ligands could help in clarifying the actual binding mode at these receptors. Comparison of different subtypes of the serotonergic receptor family (Figure S7, panels C and D) showed some differences in the arrangement of the helices. In particular, the extracellular tips of TM2, TM5 and TM7 are differently oriented towards the binding site in the 5HT_1B_ and 5HT_2B_ crystal structures. Moreover, several binding site residues vary between different subtypes. Therefore, the lack of binding of compound **1** at the 5HT_1B_ receptor could be influenced by the altered shape of the binding site due to these differences. The different conformation of TM5 observed in the 5HT_1B_ and 5HT_2B_ crystallographic structures has been previously proposed as a determinant of subtype selectivity in the serotonergic family [22].

The overall comparison of the residues involved in binding at the studied receptors revealed similarities in their ligand-binding pockets, both in terms of residue positions in the TM region and in terms of residue types among the biogenic amine receptors. Some of the residues at the corresponding positions at the ARs are also involved in the binding of nucleoside ligands, but the residue types are not conserved. Considering the high conservation of the binding residues among the biogenic amine receptors, in particular in the lower part of the binding site, we suggest that the affinity and selectivity profile of the adenosine congeners is determined by differences of residues located in the upper part of the binding site and mainly in the ELs and also by the different overall arrangement of the TM helices in each receptor that determines the actual shape of the binding cavity. These two factors can explain why the adenosine congeners do not bind all the receptors in a particular sub-family. Moreover, the proposed binding modes suggest that the pharmacophore region of the adenosine congeners particular for each off-target receptor binds deeper in the binding site, while the portions of the ligand that tune the affinity among the series interact with residues in the upper TM region and ELs. The contact with extracellular regions could affect the ligand's optimal fit in the deeper part of the cavity to interact with essential residues.

Therefore, docking studies were able to rationalize the off-target binding of the adenosine congeners at several GPCRs and highlight the ligand structural features required for the interaction to each receptor using its 3D structural information. The ability of ligand-bound AR models to serve as docking templates to select novel ligands at closely related AR subtypes was shown [31]. Now we analyze similarities between more distantly related GPCRs to reveal undetected off-target activities of compounds previously characterized as highly AR-selective. Some general factors affecting drug promiscuity and polypharmacology have been analyzed systematically using databases from pharmaceutical development, but these reports do not focus on detailed GPCR binding sites [1], [32]. Haupt et al. found that global structural and binding site similarity have a greater influence on drug promiscuity than routinely analyzed physicochemical properties or ligand flexibility [32]. Similarly, the off-target interactions detected in our study did not correlate with simple parameters such as hydrophobicity, molecular weight or other overall physicochemical properties, although the data size is very limited. Table S3 shows the physical parameters for each of the compounds **1–10** and the total number of off-target interactions determined in this study (2–11 off-target activities for each compound). There appears to be no obvious correlation between these parameters, for example molecular weight, and number of off-target interactions. These analogues are relatively rigid with only 2–5 rotatable bonds; therefore, it is not feasible to analyze the effects of ligand flexibility. The *N* ^6^-methyl derivative **5** was the least promiscuous compound of this series, with only one off-target GPCR binding site (β_3_ adrenergic receptor). This agrees with the docking observation that at many of the analyzed off-target receptors the adenine congeners bind with the *N* ^6^ substituent located in the lower part of the binding site with the amino group interacting with the conserved Asp 3.32. Therefore, truncation of the *N* ^6^-(3-chlorobenzyl) group prevents such binding mode and can explain the null affinity of compound **5** at most aminergic receptors.

Biological interactions between ARs and various other GPCRs suggest that an analysis of off-target effects of AR ligands is generally important for an understanding of the pharmacology. For example, the serotonin system is partly colocalized with the A_3_AR, and there is a regulated physical association [33]. Given the relatively high affinity of several of these congeners at 5HT receptor subtypes, there could be pharmacological implications.

Some of the observed interactions with other receptors could eventually be optimized for a beneficial therapeutic purpose. For example, activity at various neurotransmitter receptors could be synergistic with the action of A_3_AR agonists in the treatment of neuropathic pain [15]. On the other hand we have shown A_3_AR agonist **4** to be an antagonist of the 5HT_2B/2C_ receptors, an action that could be detrimental to its efficacy in pain control. Compound **9** is likely a mixed antagonist of A_3_/α_2_ receptors. Approaches to design drugs acting at multiple sites have been discussed [34]. In the future, it may be possible to adjust by design polypharmacology at GPCRs or other receptors to obtain a desired biological effect in a given compound series. The ability to predict some likely off-target interactions for analogues of this set of substructures will aid in the future structural modification of related adenosine/adenines toward therapeutic goals.

## Conclusions

We systematically examined the promiscuity of a set of adenosine/adenine congeners (recently synthesized AR ligands) to detect unanticipated interactions of these rigidified and highly substituted nucleosides and their substructures with numerous off-target sites, such as biogenic amine receptors. Adenosine receptor agonists and antagonists are now being developed as experimental drugs for cancer, inflammatory diseases, pain, glaucoma, cardiac ischemia and other diseases. Thus, the complete characterization of off-target effects of relevant nucleoside ligands is of great interest in pharmaceutical development.

Our systematic analysis of the non-AR binding interactions of this closely related series of AR ligands has allowed an understanding of the structural requirements for these off-target interactions with other Family A GPCRs. Successively truncated structures of potent AR agonists revealed pharmacophores at other receptors that could be defined in 3D by receptor docking. Although this data set is small, due to the relatedness within the set, it is possible to define required, optional and detrimental regions of the molecules with respects to some of the off-target interactions. If desired, more detailed SAR could be generated for each case, in order to enhance or eliminate that interaction while preserving the principle AR target.

Similar analyses could be performed for ligands for other GPCRs that are unrelated to these adenosine/adenine congeners. The systematic correlation of functionality on the ligands with specific amino acid residues and regions of the receptors could later be applied to predicting promiscuity of new analogues within a ligand family. Such an analysis could be useful in the drug discovery process, for guiding the design of additional structural analogues that either eliminate or accentuate certain off-target activities.

## Materials and Methods

### Pharmacological Screening

K_i_ determinations and binding profiles data of selected adenosine/adenine congeners in a broad screen of receptors and channels (including hERG) were generously provided by the National Institute of Mental Health's Psychoactive Drug Screening Program (NIMH PDSP), Contract # HHSN-271-2008-00025-C. The NIMH PDSP is directed by Bryan L. Roth MD, PhD, at the University of North Carolina at Chapel Hill and Project Officer Jamie Driscol at NIMH, Bethesda MD, USA. For experimental details, please refer to the PDSP web site http://pdsp.med.unc.edu/and click on “Binding Assay” or “Functional Assay” on the menu bar.

### Molecular Modeling

#### GPCR structures

Three-dimensional information of target GPCRs, whose structures have been solved by X-ray crystallography, was retrieved from the Protein Data Bank (PDB) [35]. For those target GPCRs lacking crystallographic structures we built homology models based on the closest available templates. To build each model, the sequence of the target receptor was retrieved from the UniProtKB database [36] and was aligned against the sequences of the structural templates available in the PDB to identify the GPCR structure with the highest similarity to be used as a template. All the alignments were performed using the software MOE [37] with the Blosum62 matrix and manually refined considering the highly conserved residues in each TM domain and allowing no gaps in the helices. Then, 3D models based on the selected GPCR template were built by means of the Homology Model tool implemented in the software MOE. After the models were built, they were subjected to energy minimization using the AMBER99 force field with a convergence threshold on the gradient of 0.01 kcal/(mol Å). We used the Protonate 3D methodology, part of the MOE suite, for protonation state assignment. The stereochemical quality of each model was checked using several tools (Ramachandran plot; backbone bond lengths, angles, and dihedral plots; clash contacts report; rotameric strain energy report) implemented in the MOE suite.

#### Molecular docking

Structures of compounds were built using the builder tool implemented in the MOE suite and subjected to energy minimization using the MMFF94x force field until a root mean square gradient of 0.05 kcal/(mol^.^Å) was obtained. Molecular docking of the ligands at the crystal structures or homology models of target GPCRs was performed by means of the Glide [38] package part of the Schrödinger suite [39]. The docking site was defined either using the co-crystallized ligand, if available, or the SiteMap [40] tool part of the Schrödinger suite. The docking grid was built using an inner box (ligand diameter midpoint box) of 10 Å×10 Å×10 Å and an outer box (box within which all the ligand atoms must be contained) that extended 20 Å in each direction from the inner one. Docking of ligands was performed in the rigid binding site using the SP (standard precision) procedure. The top scoring docking conformations for each ligand were subjected to visual inspection and analysis of the ligand-receptor interactions to select the final binding mode proposed.

## Supporting Information

Figure S1 **Representative full curves for binding inhibition of derivatives 1–10 at off-target sites.**
(PDF)

Figure S2 **Representative full curves for functional assays at selected off-target sites.**
(PDF)

Figure S3 **Alignments used for homology modeling.** Sequence alignments used to build all the homology models used in the study. Transmembrane helix regions are highlighted with orange boxes. (A) α_2B_ adrenergic receptor sequences aligned to the h5HT_1B_ crystal structure sequence (PDB ID: 4IAR), (B) α_2C_ adrenergic receptor sequences aligned to the h5HT_1B_ crystal structure sequence (PDB ID: 4IAR), (C) h5HT_2C_ serotonergic receptor sequence aligned to the h5HT_2B_ crystal structure sequence (PDB ID: 4IB4), (D) h5HT_7_ serotonergic receptor sequence aligned to the h5HT_1B_ crystal structure sequence (PDB ID: 4IAR) and (E) β_3_ adrenergic receptor sequence aligned to the β_1_ crystal structure sequence (PDB ID: 4AMJ).
(PDF)

Figure S4 **Boundaries of docking boxes.** The boundaries of the region explored for docking are highlighted for each studied receptor subtype. The docking grid was built using an inner box (ligand diameter midpoint box, boundaries shown in green) of 10 Å×10 Å×10 Å and an outer box (box within which all the ligand atoms must be contained, boundaries shown in purple) that extended 20 Å in each direction from the inner one. The highly conserved Asp 3.32 is shown in spheres in each receptor, as reference point. (A) α_2B_ model (B) α_2C_ model (C) 5HT_2B_ crystal (D) 5HT_2C_ model (E) 5HT_7_ model and (F) β_3_ model.
(PDF)

Figure S5 **Crystallographic poses of ligand complexes as determined by X-ray crystallography and results of self-docking.** Comparison of the crystallographic pose (cyan carbons) and top-scoring docking pose (yellow carbons) of: (A) the biased agonist carvedilol at the turkey β_1_ adrenergic receptor (PDB ID: 4AMJ), (B) the agonist ergotamine at the human 5HT_1B_ serotonergic receptor (PDB ID: 4IAR) and (C) the agonist ergotamine at the human 5HT_2B_ serotonergic receptor (PDB ID: 4IB4). Ligands are shown in ball and stick and some residues important for ligand recognition are shown in stick (gray carbons). Hydrogen atoms are not displayed. H-bonds and salt bridges are shown as black dashed lines. The Connolly surface of the amino acids surrounding the binding site is displayed. Surface color indicates the lipophilic potential: lipophilic regions (green), neutral regions (white) and hydrophilic regions (magenta).
(PDF)

Figure S6 **Docking of known aminergic ligands at target receptors.** Results of docking studies performed for known aminergic ligands at selected target receptors (models or crystal structures). Binding modes proposed for: (A) the agonist noradrenaline (cyan carbons) and the antagonist spiroxatrine (magenta carbons) at the human α_2B_ adrenergic receptor model, (B) the antagonists carvedilol (magenta carbons), bupranolol (cyan carbons) and nadolol (yellow carbons) at the human β_3_ adrenergic receptor model and (C) the agonist serotonin (cyan carbons) and the antagonist EGIS-7625 (magenta carbons) at the human 5HT_2B_ serotonergic receptor (PDB ID: 4IB4). Ligands are shown in ball and stick and some residues important for ligand recognition are shown in stick (gray carbons). Hydrogen atoms are not displayed.
(PDF)

Figure S7 **Comparison of receptors within the same subfamily.** (A) Side view and (B) top view of the superposition of turkey β_1_ adrenergic receptor (PDB ID: 4AMJ) (green carbons), human β_2_ adrenergic receptor (PDB ID: 2RH1) (cyan carbons) and human β_3_ adrenergic receptor model (pink carbons). The second proposed docking pose of compound **3** (orange carbons) as an example at the human β_3_ adrenergic receptor model is displayed. For the β_3_ adrenergic receptor residues at 4 Å from the ligand are displayed. For the β_1_ and β_2_ adrenergic receptors only residues at 4 Å from the ligand that differ from the β_3_ subtype are displayed. (C) Side view of the superposition of human 5HT_1B_ serotonergic receptor (PDB ID: 4IAR) (green carbons), human 5HT_2B_ serotonergic receptor (PDB ID: 4IB4) (pink carbons), human 5HT_2C_ serotonergic receptor model (orange carbons) and human 5HT_7_ serotonergic receptor model (cyan carbons). (D) top view of the superposition of human 5HT_1B_ serotonergic receptor (green carbons) and human 5HT_2B_ serotonergic receptor (pink carbons). In C and D the proposed docking pose of compound **1** (yellow carbons) as an example at the human 5HT_2B_ serotonergic receptor is displayed. For the 5HT_2B_ serotonergic receptor residues at 4 Å from the ligand are displayed. For the 5HT_1B_, 5HT_2C_ and 5HT_7_ serotonergic receptors only residues at 4 Å from the ligand that differ from the 5HT_2B_ subtype are displayed.
(PDF)

Table S1 **Binding activity of the adenosine/adenine derivatives 1–10 at three subtypes of human ARs.**
(PDF)

Table S2 **Percent inhibition of radioligand binding of the adenosine/adenine derivatives 1–10 in binding to off-target GPCRs, ion channels and a transporter.**
(PDF)

Table S3 **Physical parameters for each of the adenosine/adenine derivatives 1–10.** The total number of off-target interactions (including σ receptors, PBR and one ion channel: 5HT_3_) determined (in binding assays, unless noted) in this study is indicated.
(PDF)

Text S1 **List of all the binding sites for which the primary screening at 10 µM was performed.**
(PDF)

PDB File S1 **3D coordinates of the first proposed docking mode of compound 4 at the human 5HT_2B_ serotonergic receptor crystal structure.**
(PDB)

PDB File S2 **3D coordinates of the second proposed docking mode of compound 4 at the human 5HT_2B_ serotonergic receptor crystal structure.**
(PDB)
