# Supplementary material for: Structural Probing of Off-Target G Protein-Coupled Receptor Activities within a Series of Adenosine/Adenine Congeners
Source: PLoS One. 2014 May 23;9(5):e97858. doi: 10.1371/journal.pone.0097858 (PMC4032265; doi:10.1371/journal.pone.0097858)

Figure S1. Representative full curves for binding inhibition of derivatives 1-10 at off-target sites.

Compound 1 (MRS5698)

α_{2A}

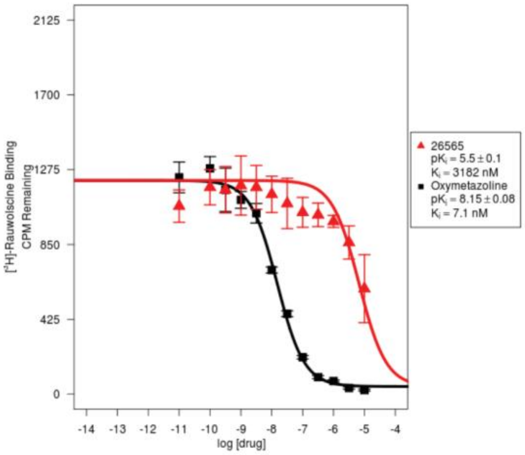

β_3

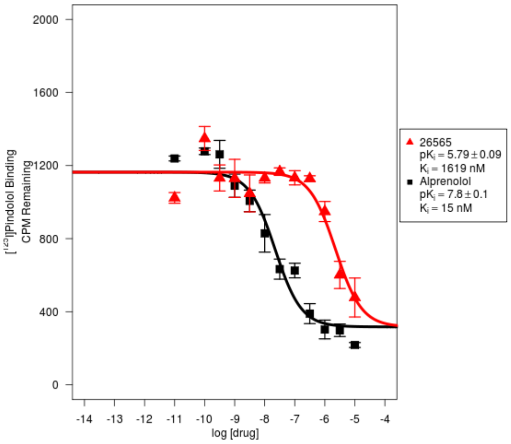

α_{2B}

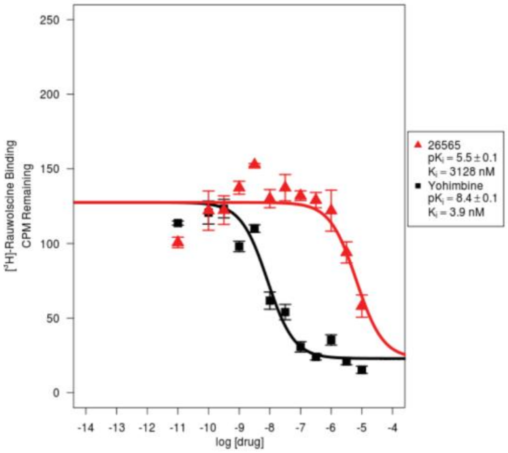

DOR

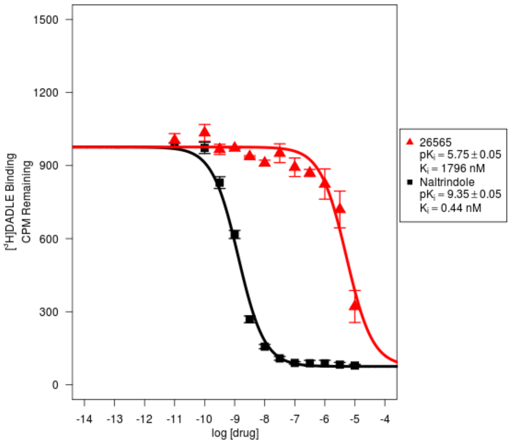

α_{2C}

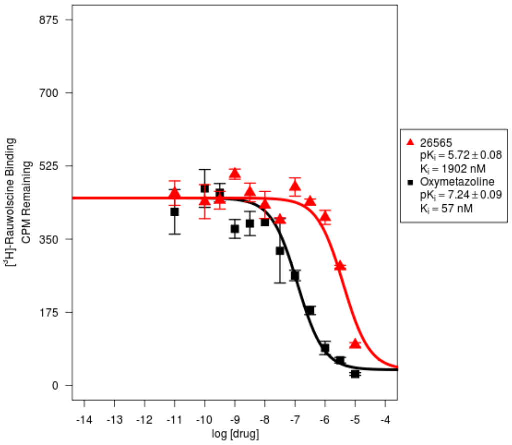

$5HT_{1A}$

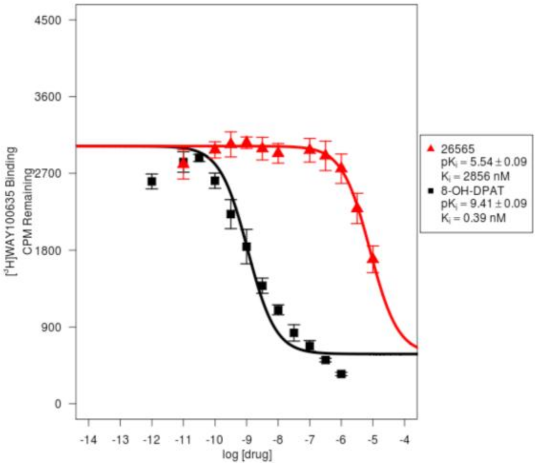

$5HT_{2B}$

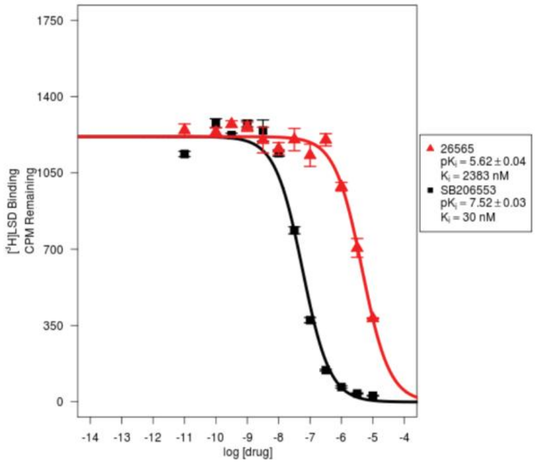

PBR

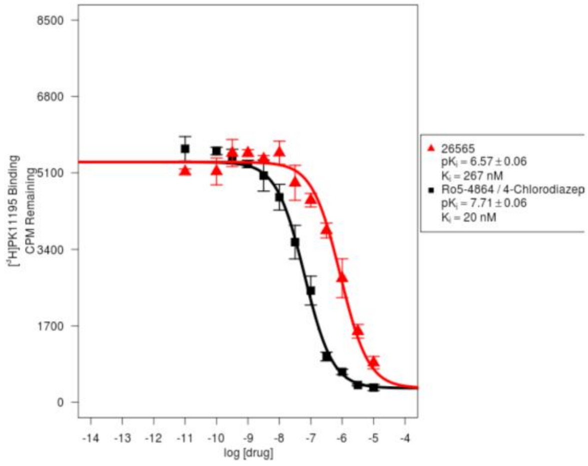

$5HT_{2C}$

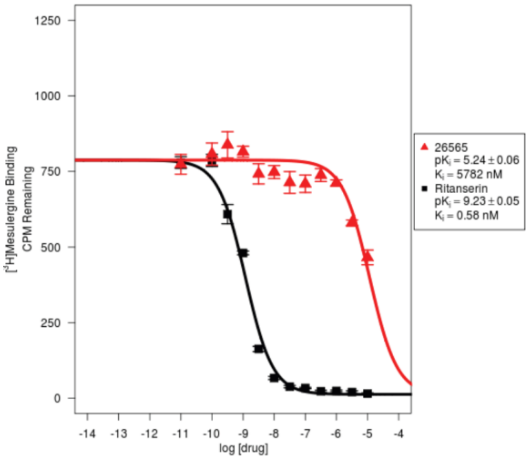

σ2

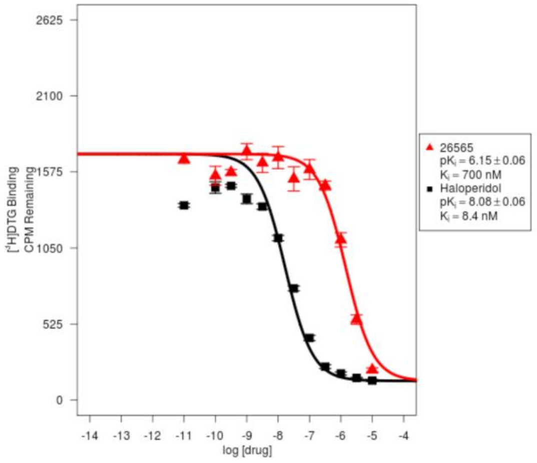

Compound 2 (MRS5678)

α_{2C}

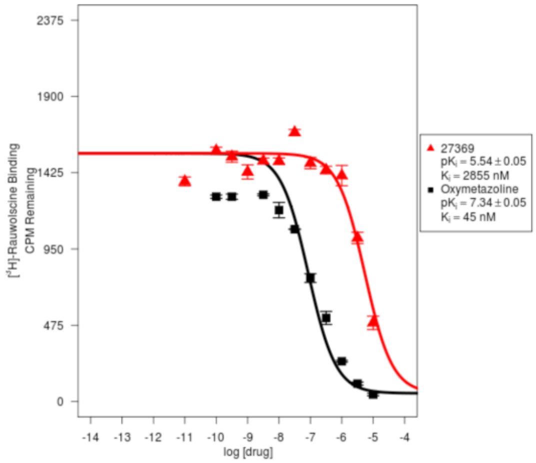

DOR

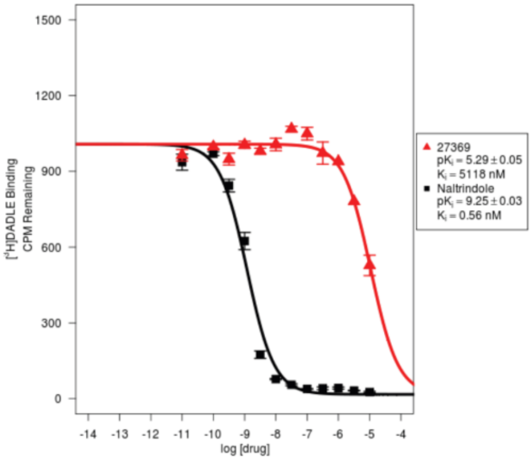

β_3

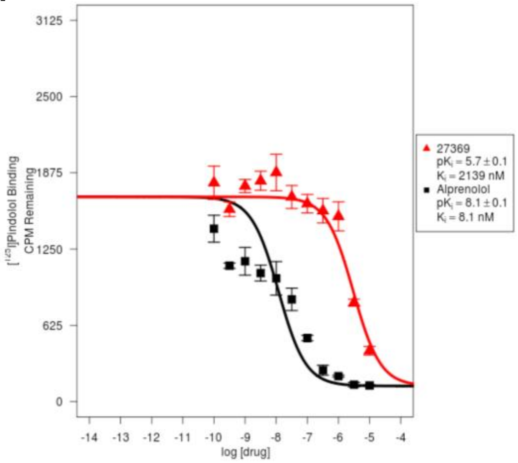

PBR

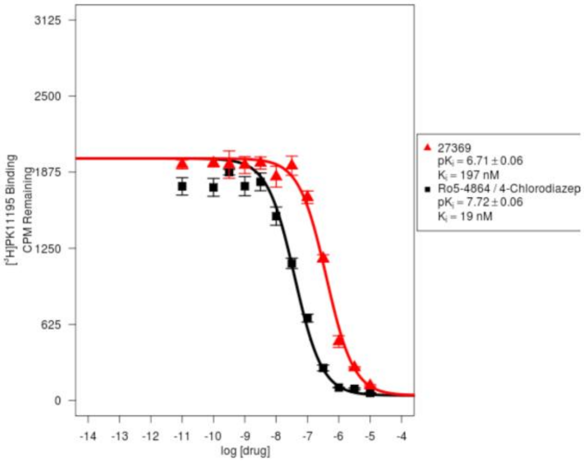

$5HT_{2B}$

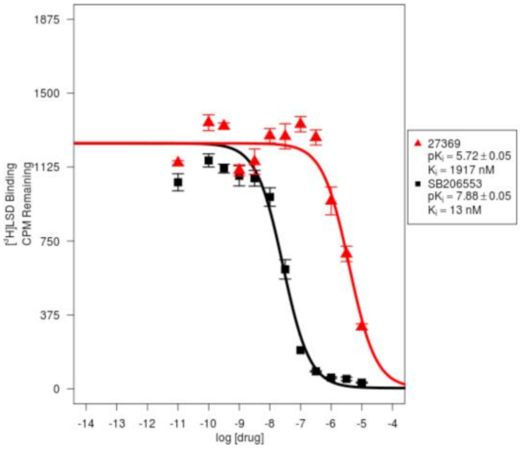

Compound 3 (MRS5697)

α_{2B}

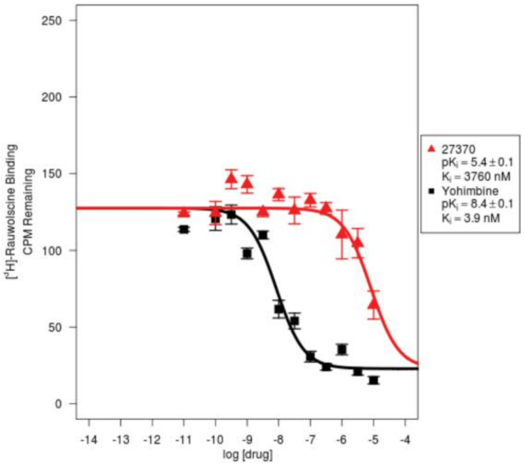

$5HT_{2B}$

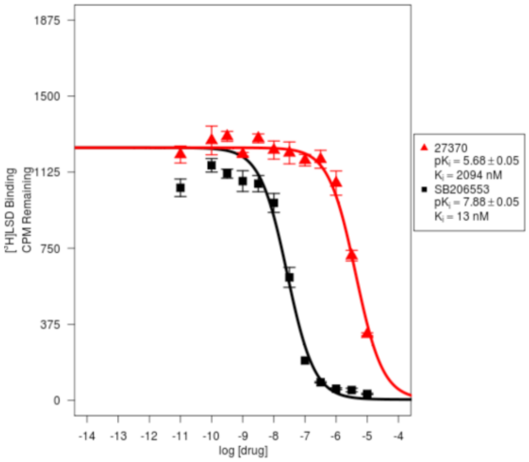

α_{2C}

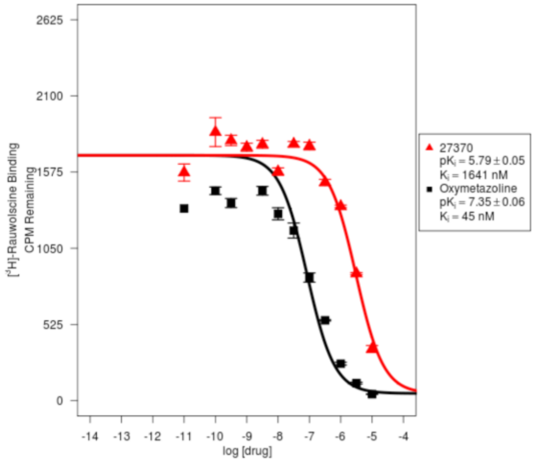

$5HT_{5A}$

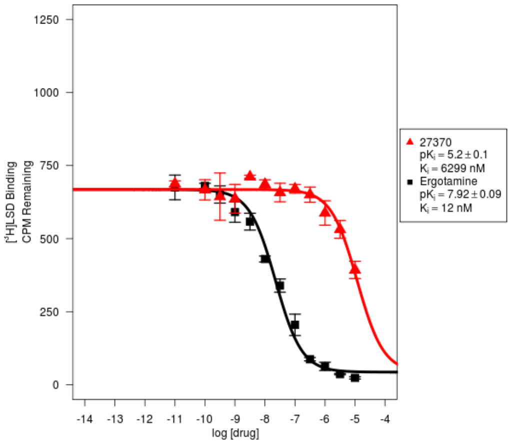

β_3

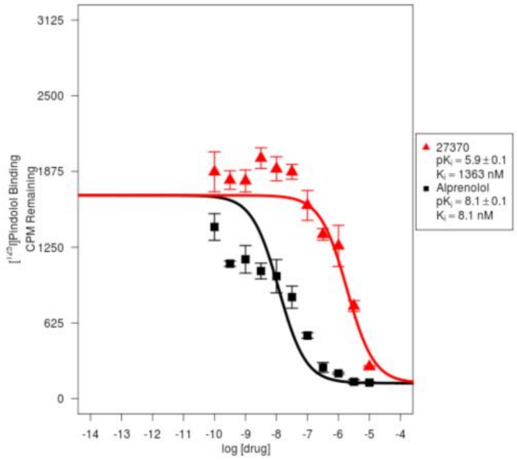

PBR

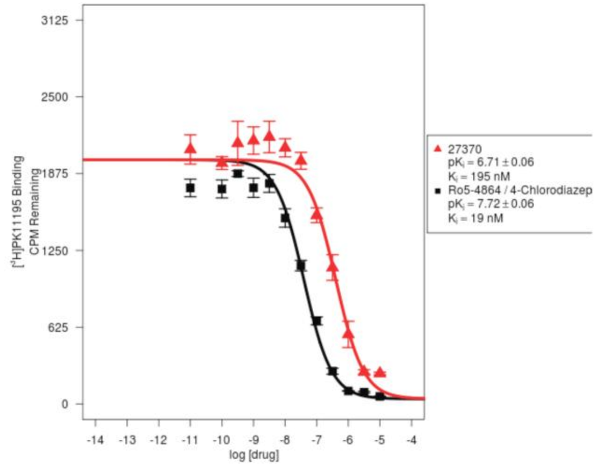

Compound 4 (MRS3558)

$5HT_{2B}$

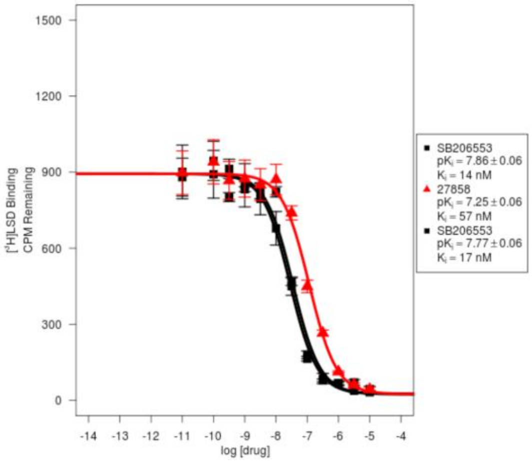

$5HT_{2C}$

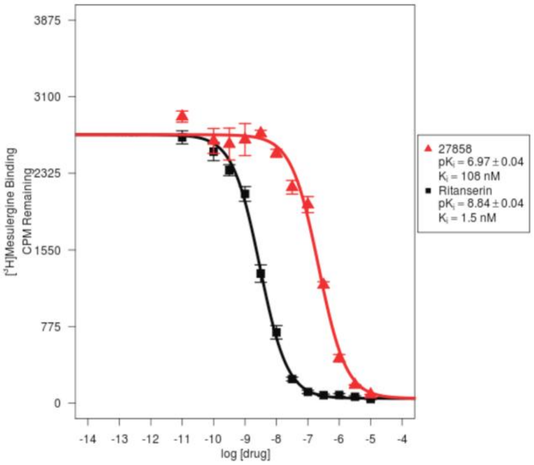

$5HT_7$

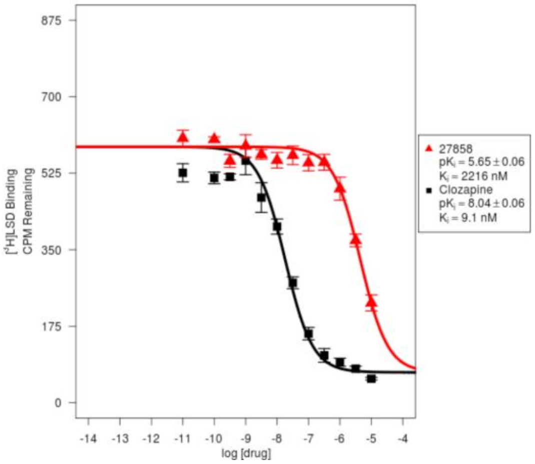

Compound 5 (MRS5676)

β_3

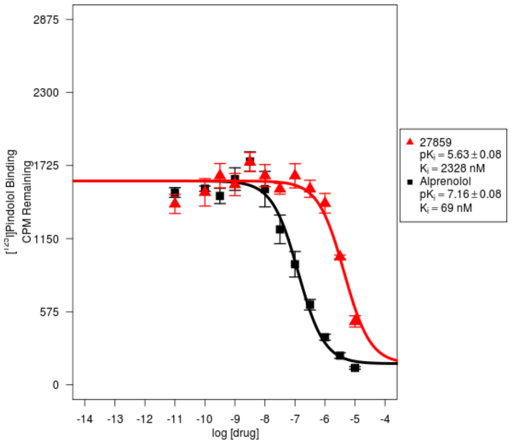

Compound 6 (MRS5755)

α_{2B}

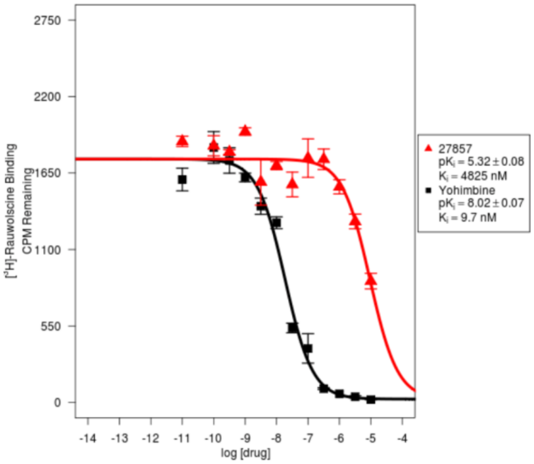

σ2

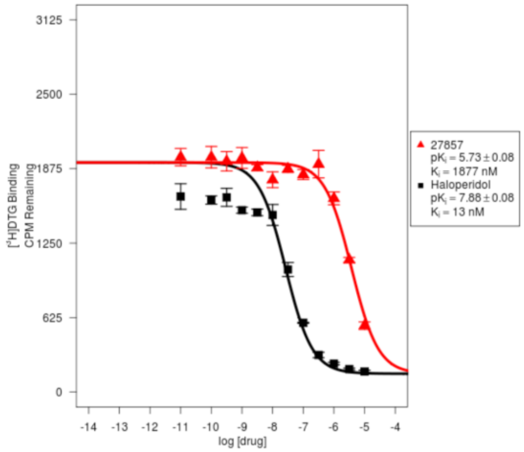

α_{2C}

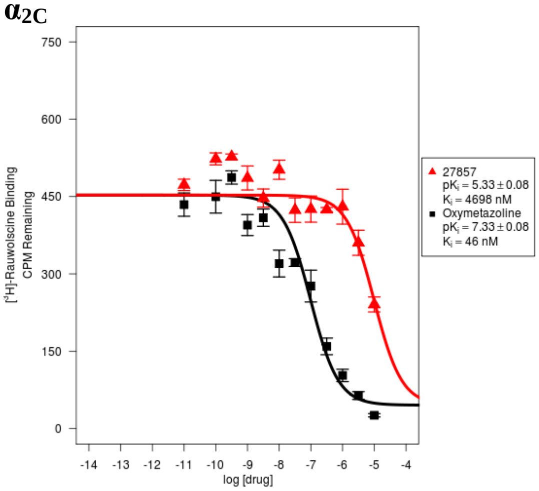

PBR

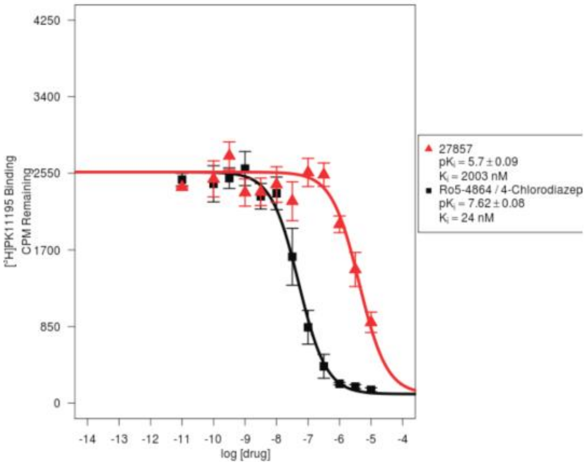

β_3

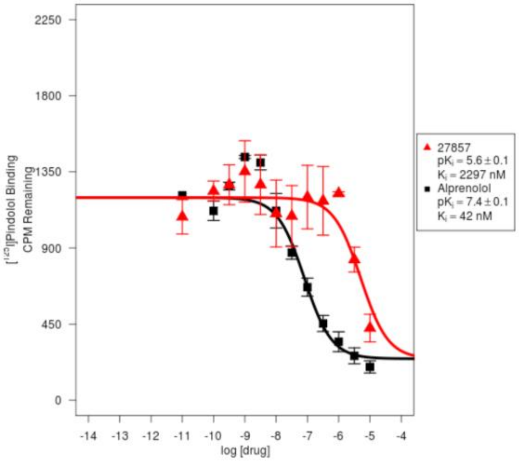

Compound 7 (MRS5202)

α_{2B}

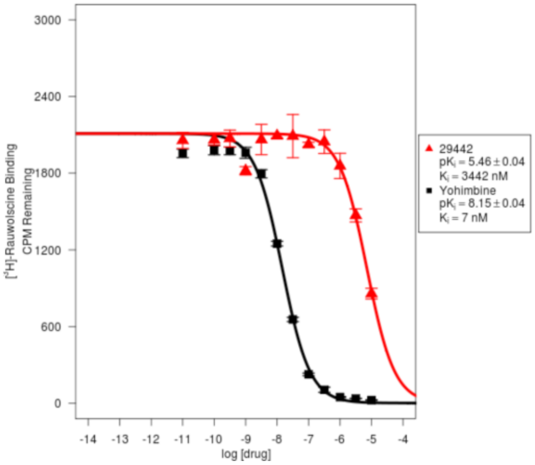

$5HT_{2C}$

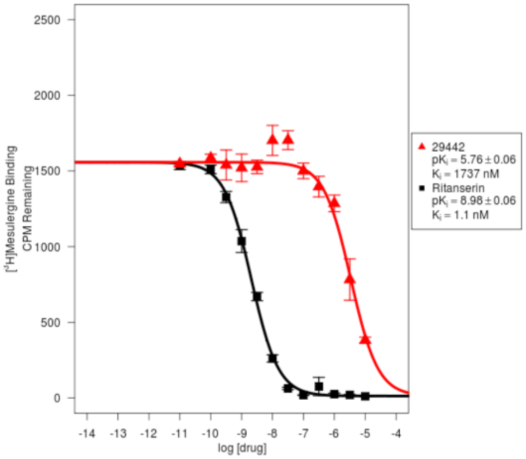

α_{2C}

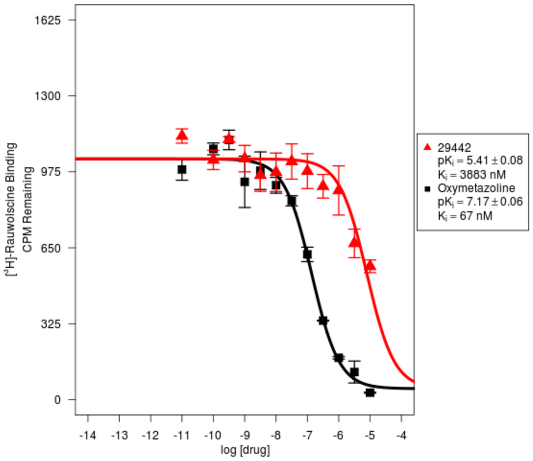

$5HT_3$

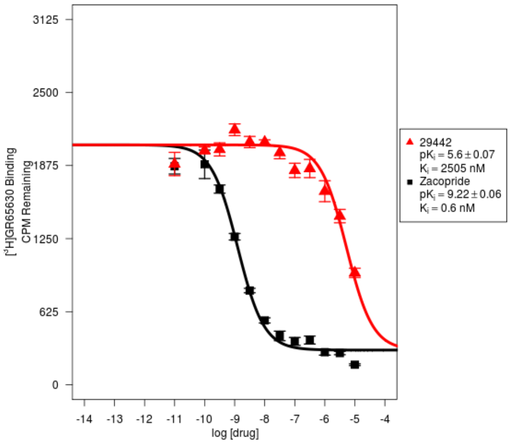

$5HT_{2B}$

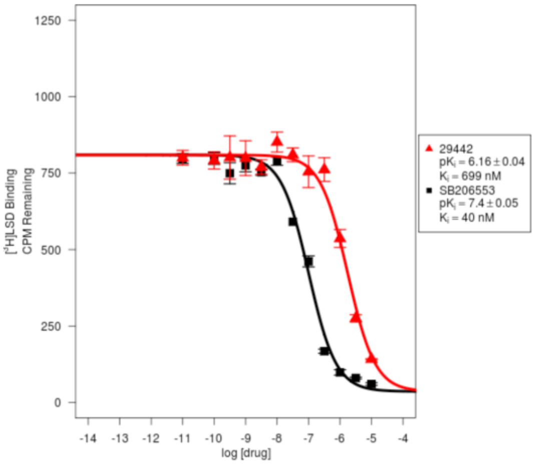

$5HT_7$

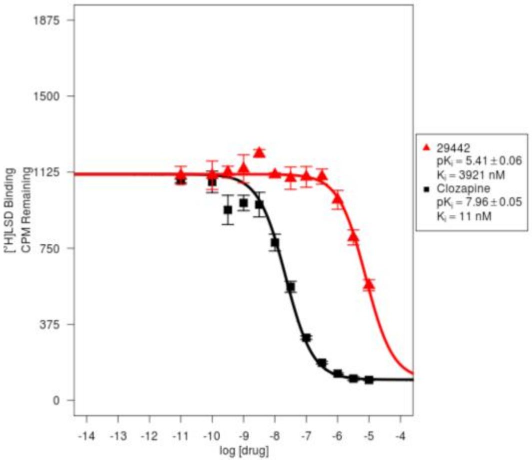

σ2

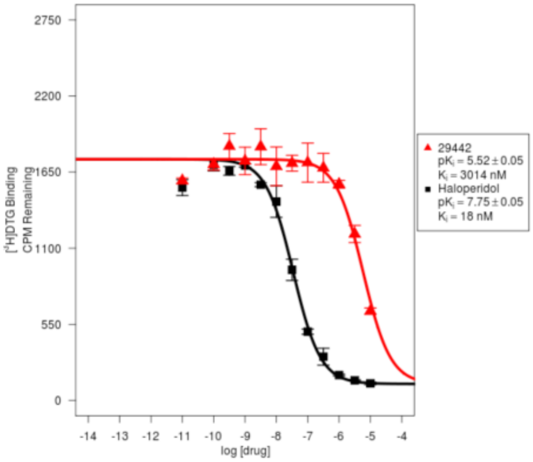

[³H]DTG Binding
CPM Remaining
2750
2200
1650
1100
550
0
-14 -13 -12 -11 -10 -9 -8 -7 -6 -5 -4
log [drug]
29442
pKi = 5.52 ± 0.05
Ki = 3014 nM
Haloperidol
pKi = 7.75 ± 0.05
Ki = 18 nM

Compound 8 (MRS5923)

α_{2A}

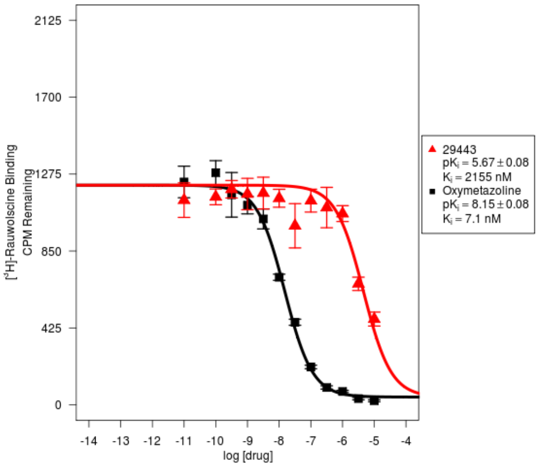

$5HT_{2B}$

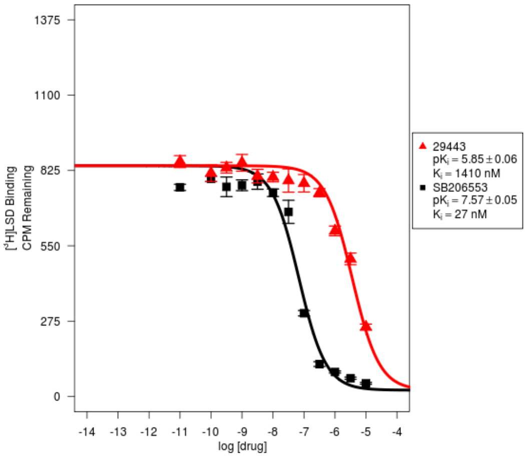

α_{2B}

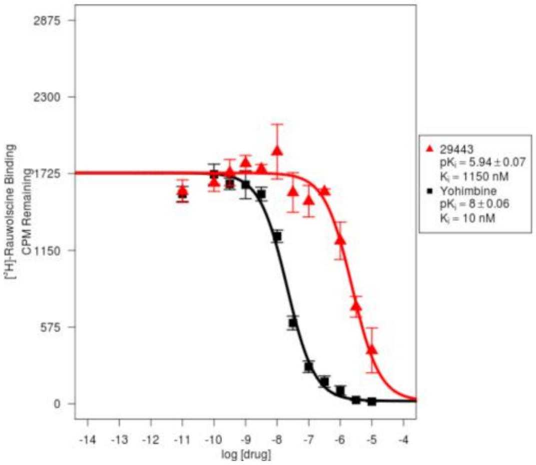

σ_1

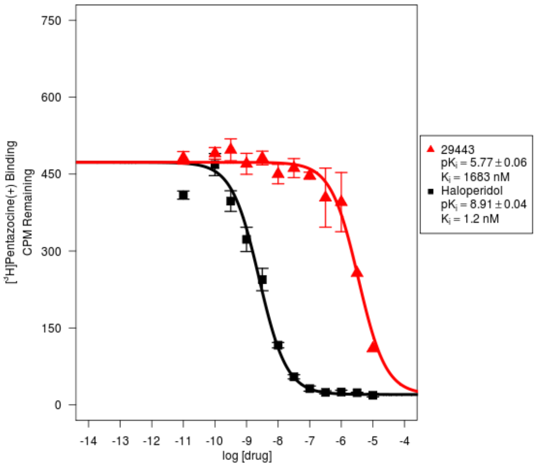

α_{2C}

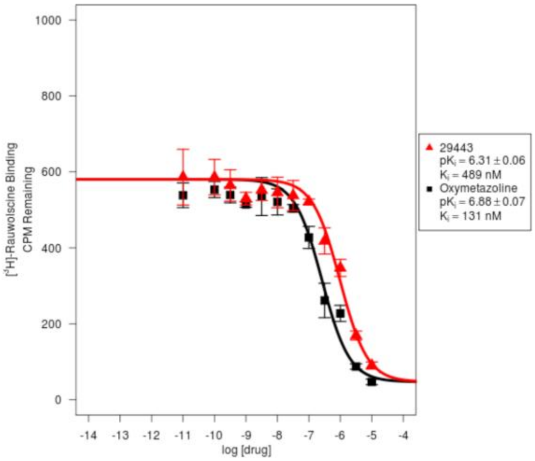

Compound 9 (MRS5930)

α_{2A}

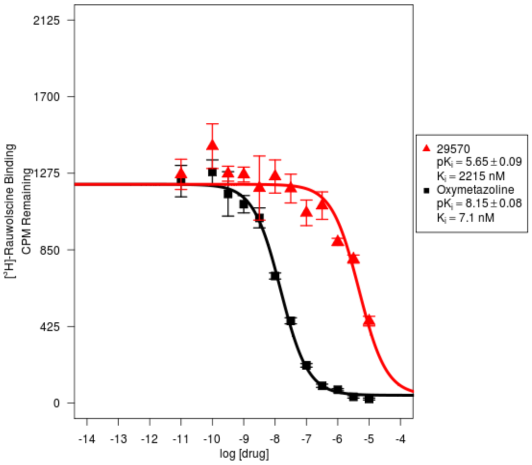

5HT$_{2B}$

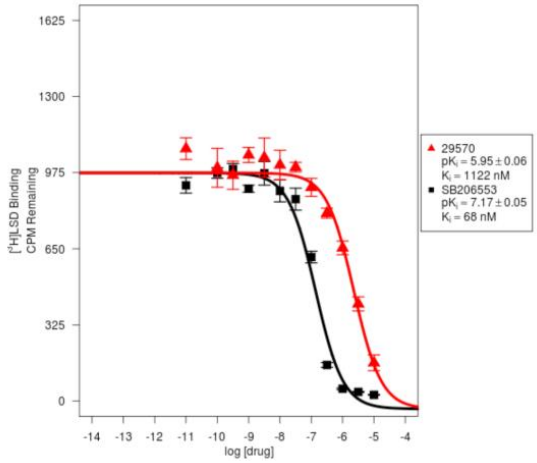

α_{2B}

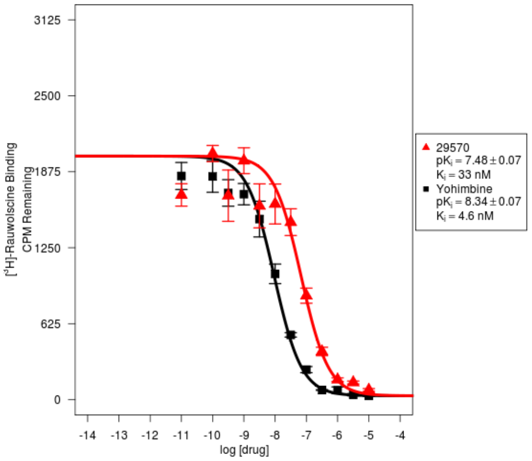

5HT$_{2C}$

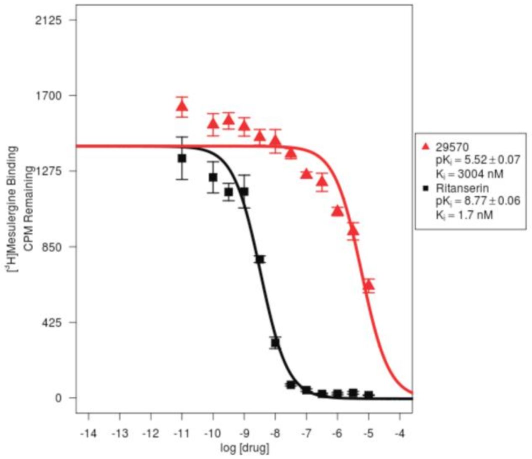

5HT$_{5A}$

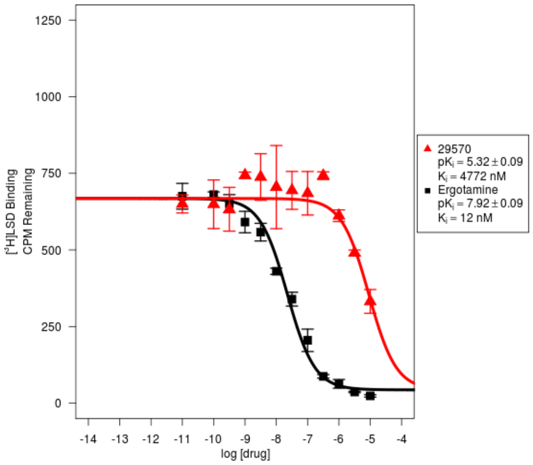

α_{2C}

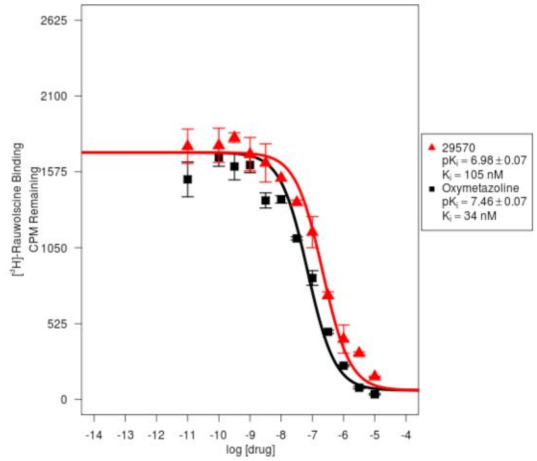

Compound 10 (MRS5474)

$5HT_{2B}$

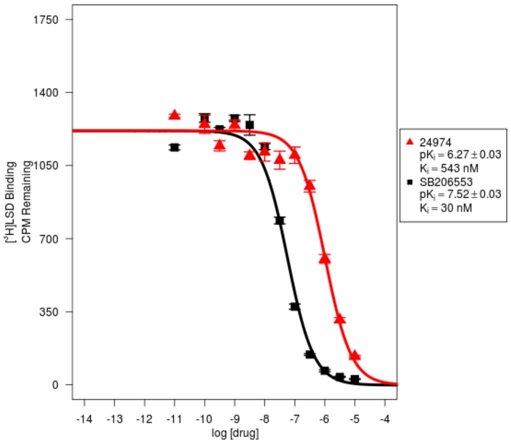

$5HT_{2C}$

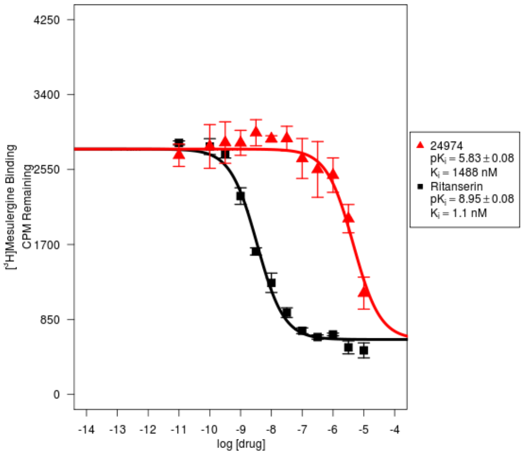

Supplement: Figure S1 — Representative full curves for binding inhibition of derivatives 1–10 at off-target sites. (PDF) [file pone.0097858.s001.pdf]
