# Supplementary material for: Structural Probing of Off-Target G Protein-Coupled Receptor Activities within a Series of Adenosine/Adenine Congeners
Source: PLoS One. 2014 May 23;9(5):e97858. doi: 10.1371/journal.pone.0097858 (PMC4032265; doi:10.1371/journal.pone.0097858)

Figure S2. Representative full curves for functional assays at selected off-target sites.

Data were determined by the PDSP. Full procedures are available online at the PDSP web site http://pdsp.med.unc.edu/.

Assay showing lack of agonist activity of **4** (29844) and **9** (29570) at $5HT_{2B}$ and $5HT_{2C}$ receptors in comparison to agonist serotonin (5HT). IC_{50} values of **4** and **9** at $5HT_{2B}$ receptors were 3.26 and 0.89 μM, respectively. IC_{50} value of **9** at the $5HT_{2C}$ receptor was ~0.8 μM.

IC_{50} value of **9** at the α_{2C} adrenergic receptor was determined to be 2.9 μM.

Thus, all of these interactions are as antagonist.

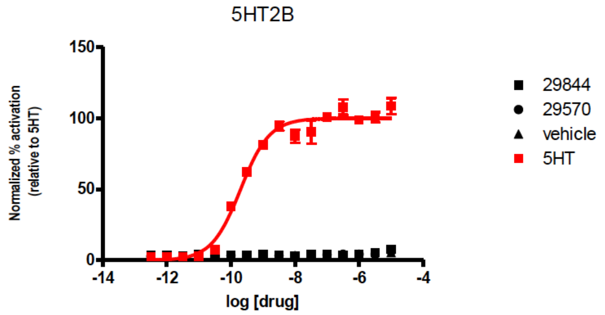

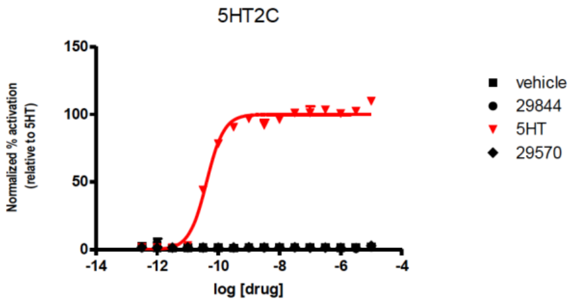

Nicotinic acetylcholine receptor antagonist activity: Compound **10** inhibited $^{86}Rb^{+}$ efflux from cells expressing the $KX\alpha_3\beta_4$ receptor 2 with an IC_{50} of 21.2 μM and from cells expressing the $YX\alpha_3\beta_4H1$ receptor with an IC_{50} of 30.3 μM.

There was no inhibition of $[^3H]$epibatidine binding in cells expressing the $KX\alpha_4\beta_2$ receptor 2.

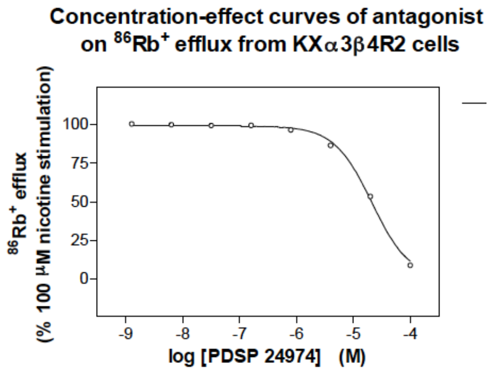

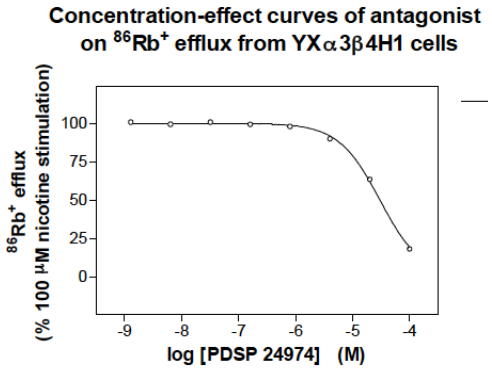

Supplement: Figure S2 — Representative full curves for functional assays at selected off-target sites. (PDF) [file pone.0097858.s002.pdf]
