# Supplementary material for: Structural Probing of Off-Target G Protein-Coupled Receptor Activities within a Series of Adenosine/Adenine Congeners
Source: PLoS One. 2014 May 23;9(5):e97858. doi: 10.1371/journal.pone.0097858 (PMC4032265; doi:10.1371/journal.pone.0097858)
Supplement: Figure S3 — Alignments used for homology modeling. Sequence alignments used to build all the homology models used in the study. Transmembrane helix regions are highlighted with orange boxes. (A) α2B adrenergic receptor sequences aligned to the h5HT1B crystal structure sequence (PDB ID: 4IAR), (B) α2C adrenergic receptor sequences aligned to the h5HT1B crystal structure sequence (PDB ID: 4IAR), (C) h5HT2C serotonergic receptor sequence aligned to the h5HT2B crystal structure sequence (PDB ID: 4IB4), (D) h5HT7 serotonergic receptor sequence aligned to the h5HT1B crystal structure sequence (PDB ID: 4IAR) and (E) β3 adrenergic receptor sequence aligned to the β1 crystal structure sequence (PDB ID: 4AMJ). (PDF) [file pone.0097858.s003.pdf]

Figure S3. Alignments used for homology modeling. Sequence alignments used to build all the homology models used in the study. Transmembrane helix regions are highlighted with orange boxes. (A) α_{2B} adrenergic receptor sequences aligned to the $h5HT_{1B}$ crystal structure sequence (PDB ID: 4IAR), (B) α_{2C} adrenergic receptor sequences aligned to the $h5HT_{1B}$ crystal structure sequence (PDB ID: 4IAR), (C) $h5HT_{2C}$ serotonergic receptor sequence aligned to the $h5HT_{2B}$ crystal structure sequence (PDB ID: 4IB4), (D) $h5HT_7$ serotonergic receptor sequence aligned to the $h5HT_{1B}$ crystal structure sequence (PDB ID: 4IAR) and (E) β_3 adrenergic receptor sequence aligned to the β_1 crystal structure sequence (PDB ID: 4AMJ).

A

```
alpha2B (P18089)    1  ~~~~~~~~MDHQDPYSVQATAAIAAAITFLILFTIFGNALVILAVLTSRSLRAPQNLF 50
5HT1B (4IAR)        9  ~~~~~~~~YIYQDSISLPWKVLLVMLLALITLATTLSNAFVIATVYRTRKLHTPANYL 58

alpha2B (P18089)   51  LVSLAAADILVATLIIPFSLANELLGYWYFRRTWCEVYLALDVLFCTSSIVHLCAISL 108
5HT1B (4IAR)       59  IASLAVTDLLVSILVMPISTMYTVTGRWTLGQVVCDFWLSSDITCCTASIWHLCVIAL 116

alpha2B (P18089)  109  DRYWAVSRALEYNSKRTPRRIKCIILTVWLIAAVISLPPLIYKGDQGPQPRGRPQCKL 166
5HT1B (4IAR)      117  DRYWAITDAVEYSAKRTPKRAAVMIALVWVFSISISLPPFFWR~~QAKAEEEVSECVV 172

alpha2B (P18089)  167  NQE~AWYILASSIGSFFAPCLIMILVYLRIYLIAKRSNRRGPRAKGGPGQGESKQPRP 223
5HT1B (4IAR)      173  NTDHILYTVYSTVGAFYFPTLLLIALYGRIYVEARSRI~~~~~~~~~~~~~~~~~~~~ 210

alpha2B (P18089)  224  DHGGALASAKLPALASVASAREVNGHSKSTGEKEEGETPEDTGTRALPPSWAALPNSG 281
5HT1B (4IAR)           ~~~~~~~~~~~~~~~~~~~~~~~~~~~~~~~~~~~~~~~~~~~~~~~~~~~~~~~~~~

alpha2B (P18089)  282  QGQKEGVCGASPEDEAEEEEEEEEEEEECEPQAVPVSPASACSPPLQQPQGSRVLATL 339
5HT1B (4IAR)           ~~~~~~~~~~~~~~~~~~~~~~~~~~~~~~~~~~~~~~~~~~~~~~~~~~~~~~~~~~

alpha2B (P18089)  340  RGQVLLGRGVGAIGGQWWRRRAQLTREKRFTFVLAVVIGVFVLCWFPFFFSYSLGAIC 397
5HT1B (4IAR)      211  ~~~~~~~~~~~~~~~~~~~~~~~AARERKATKTLGIILGAFIVCWLPFFIISLVMPIC 245

alpha2B (P18089)  398  PKHCKVPHGLFQFFFWIGYCNSSLNPVIYTIFNQDFRRAFRRILCRPWTQTAW 450
5HT1B (4IAR)      246  KDACWFHLAIFDFFTWLGYLNSLINPIIYTMSNEDFKQAFHKLIRFK~~~~~~ 292
```

B

```
alpha2C (P18825)    1  MASPALAAALAVAAAAGPNASGAGERGSGGVANASGASWGPPRGQYSAGAVAGLAAVV 58
5HT1B (4IAR)        9  ~~~~~~~~~~~~~~~~~~~~~~~~~~~~~~~~~~~~~~~YIYQDSISLPWKVLLVMLL 27

alpha2C (P18825)   59  GFLIVFTVVGNVLVVIAVLTSRALRAPQNLFLVSLASADILVATLVMPFSLANELMAY 116
5HT1B (4IAR)       28  ALITLATTLSNAFVIATVYRTRKLHTPANYLIASLAVTDLLVSILVMPISTMYTVTGR 85

alpha2C (P18825)  117  WYFGQVWCGVYLALDVLFCTSSIVHLCAISLDRYWSVTQAVEYNLKRTPRRVKATIVA 174
5HT1B (4IAR)       86  WTLGQVVCDFWLSSDITCCTASIWHLCVIALDRYWAITDAVEYSAKRTPKRAAVMIAL 143

alpha2C (P18825)  175  VWLISAVISFPPLVSLYRQPDGAAYPQCGLN~DETWYILSSCIGSFFAPCLIMGLVYA 231
5HT1B (4IAR)      144  VWVFSISISLPPFFWRQAKAE~EEVSECVVNTDHILYTVYSTVGAFYFPTLLLIALYG 200

alpha2C (P18825)  232  RIYRVAKLRTRTVAQAREKRFTFVLAVVMGVFVLCWFPFFFSYSLYGICREACQVPGP 289
5HT1B (4IAR)      201  RIYVEARSRI~~~~AARERKATKTLGIILGAFIVCWLPFFIISLVMPICKDACWFHLA 254

alpha2C (P18825)  290  LFKFFFWIGYCNSSLNPVIYTVFNQDFRRSFKHILFRRRRRGFRQ 334
5HT1B (4IAR)      255  IFDFFTWLGYLNSLINPIIYTMSNEDFKQAF~HKLIRFK~~~~~~ 292
```

C

```
h5HT2C (P28335)     1  MVNLRNAVHSFLVHLIGLLVWQCDISVSPVAAIVTDIFNTSDGGRFKFPDGVQNWPAL 58
h5HT2B (4IB4)      23  ~~~~~~~~~~~~~~~~~~~~~~~~~~~~~~~~~~~~~~~~~~~~~~EEQGNKLHWAAL 34

h5HT2C (P28335)    59  SIVIIIIMTIGGNILVIMAVSMEKKLHNATNYFLMSLAIADMLVGLLVMPLSLLAILY 116
h5HT2B (4IB4)      35  LILMVIIPTIGGNTLVILAVSLEKKLQYATNYFLMSLAVADLLVGLFVMPIALLTIMF 92

h5HT2C (P28335)   117  DYVWPLPRYLCPVWISLDVLFSTASIMHLCAISLDRYVAIRNPIEHSRFNSRTKAIMK 174
h5HT2B (4IB4)      93  EAMWPLPLVLCPAWLFLDVLFSTASIWHLCAISVDRYIAIKKPIQANQYNSRATAFIK 150

h5HT2C (P28335)   175  IAIVWAISIGVSVPIPVIGLRDEEKVFVNNTTCVLND~~~PNFVLIGSFVAFFIPLTI 229
h5HT2B (4IB4)     151  ITVVWLISIGIAIPVPIKGIETDVDN~PNNITCVLTKERFGDFMLFGSLAAFFTPLAI 207

h5HT2C (P28335)   230  MVITYCLTIYVLRRQALMLLHGHTEEPPGLSLDFLKCCKRNTAEEENSANPNQDQNAR 287
h5HT2B (4IB4)     208  MIVTYFLTIHALQKKA~~~~~~~~~~~~~~~~~~~~~~~~~~~~~~~~~~~~~~~~~~ 223

h5HT2C (P28335)   288  RRKKKERRPRGTMQAINNERKASKVLGIVFFVFLIMWCPFFITNILSVLCEKSCNQKL 345
h5HT2B (4IB4)     224  ~~~~~~~~~~~~~QTISNEQRASKVLGIVFFLFLLMWCPFFITNITLVLCD~SCNQTT 267

h5HT2C (P28335)   346  MEKLLNVFVWIGYVCSGINPLVYTLFNKIYRRAFSNYLRCNYKVEKKPPVRQIPRVAA 403
h5HT2B (4IB4)     268  LQMLLEIFVWIGYVSSGVNPLVYTLFNKTFRDAFGRYITCNYRA~~~~~~~~~~~~~~ 311

h5HT2C (P28335)   404  TALSGRELNVNIYRHTNEPVIEKASDNEPGIEMQVENLELPVNPSSVVSERISSV 458
h5HT2B (4IB4)          ~~~~~~~~~~~~~~~~~~~~~~~~~~~~~~~~~~~~~~~~~~~~~~~~~~~~~~~
```

D

```
h5HT7 (P34969)      1  MMDVNSSGRPDLYGHLRSFLLPEVGRGLPDLSPDGGADPVAGSWAPHLLSEVTASPAP 58
h5HT1B (4IAR)          ~~~~~~~~~~~~~~~~~~~~~~~~~~~~~~~~~~~~~~~~~~~~~~~~~~~~~~~~~~

h5HT7 (P34969)     59  TWDAPPDNASGCGEQINYGRVEKVVIGSILTLITLLTIAGNCLVVISVCFVKKLRQPS 116
h5HT1B (4IAR)       9  ~~~~~~~~~~~YIYQDSISLPWKVLLVMLLALITLATTLSNAFVIATVYRTRKLHTPA 55

h5HT7 (P34969)    117  NYLIVSLALADLSVAVAVMPFVSVTDLIGGKWIFGHFFCNVFIAMDVMCCTASIMTLC 174
h5HT1B (4IAR)      56  NYLIASLAVTDLLVSILVMPISTMYTVTG~RWTLGQVVCDFWLSSDITCCTASIWHLC 112

h5HT7 (P34969)    175  VISIDRYLGITRPLTYPVRQNGKCMAKMILSVWLLSASITLPPLFGWAQNVNDDKV~~ 230
h5HT1B (4IAR)     113  VIALDRYWAITDAVEYSAKRTPKRAAVMIALVWVFSISISLPPFF~WRQAKAEEEVSE 169

h5HT7 (P34969)    231  CLISQD~FGYTIYSTAVAFYIPMSVMLFMYYQIYKAARKSAAKHKFPGFPRVEPDSVI 287
h5HT1B (4IAR)     170  CVVNTDHILYTVYSTVGAFYFPTLLLIALYGRIYVEARSRI~~~~~~~~~~~~~~~~~ 210

h5HT7 (P34969)    288  ALNGIVKLQKEVEECANLSRLLKHERKNISIFKREQKAATTLGIIVGAFTVCWLPFFL 345
h5HT1B (4IAR)     211  ~~~~~~~~~~~~~~~~~~~~~~~~~~~~~~~AARERKATKTLGIILGAFIVCWLPFFI 237

h5HT7 (P34969)    346  LSTARPFICGTSCSCIPLWVERTFLWLGYANSLINPFIYAFFNRDLRTTYRSLLQCQY 403
h5HT1B (4IAR)     238  ISLVMP~ICKDAC~WFHLAIFDFFTWLGYLNSLINPIIYTMSNEDFKQAFHKLIRFK~ 292

h5HT7 (P34969)    404  RNINRKLSAAGMHEALKLAERPERPEFVLRACTRRVLLRPEKRPPVSVWVLQSPDHHN 461
h5HT1B (4IAR)          ~~~~~~~~~~~~~~~~~~~~~~~~~~~~~~~~~~~~~~~~~~~~~~~~~~~~~~~~~~

h5HT7 (P34969)    462  WLADKMLTTVEKKVMIHD 479
h5HT1B (4IAR)          ~~~~~~~~~~~~~~~~~~
```

E

```
beta3 (P13945)      1  MAPWPHENSSLAPWPDLPTLAPNTANTSGLPGVPWEAALAGALLALAVLATVGGNLLV 58
beta1 (4AMJ)        2  ~~~~~~~~~~~~~~~~~~~~~~~~~~GAELLSQQWEAGMS~LLMALVVLLIVAGNVLV 32

beta3 (P13945)     59  IVAIAWTPRLQTMTNVFVTSLAAADLVMGLLVVPPAATLALTGHWPLGATGCELWTSV 116
beta1 (4AMJ)       33  IAAIGSTQRLQTLTNLFITSLACADLVVGLLVVPFGATLVVRGTWLWGSFLCELWTSL 90

beta3 (P13945)    117  DVLCVTASIETLCALAVDRYLAVTNPLRYGALVTKRCARTAVVLVWVVSAAVSFAPIM 174
beta1 (4AMJ)       91  DVLCVTASIETLCVIAIDRYLAITSPFRYQSLMTRARAKVIICTVWAISALVSFLPIM 148

beta3 (P13945)    175  SQWWRVGADAEAQRCHSNPRCCAFASNMPYVLLSSSVSFYLPLLVMLFVYARVFVVAT 232
beta1 (4AMJ)      149  MHWWR~DEDPQALKCYQDPGCCDFVTNRAYAIASSIISFYIPLLIMIFVALRVYREAK 205

beta3 (P13945)    233  RQLRLLRGELGRFPPEESPPAPSRSLAPAPVGTCAPPEGVPACGRRPARLLPLREHRA 290
beta1 (4AMJ)      206  EQIRKIDR~~~~~~~~~~~~~~~~~~~~~~~~~~~~~~~~~ASKRKTSRVMLMREHKA 230

beta3 (P13945)    291  LCTLGLIMGTFTLCWLPFFLANVLRALGGPSLVPGPAFLALNWLGYANSAFNPLIYCR 348
beta1 (4AMJ)       231  LKTLGIIMGVFTLCWLPFFLVNIVNVFNR~DLVPDWLFVAFNWLGYANSAMNPIIYCR 287

beta3 (P13945)    349  SPDFRSAFRRLLCRCGRRLPPEPCAAARPALFPSGVPAARSSPAQPRLCQRLDGASWG 406
beta1 (4AMJ)      288  SPDFRKAFKRLLA~~~~~~~~~~~~~~~~~~~~~~~~~~~~~~~~~~~~~~~~~~~~~ 300

beta3 (P13945)    407  VS 408
beta1 (4AMJ)           ~~
```
