# Supplementary material for: Structural Probing of Off-Target G Protein-Coupled Receptor Activities within a Series of Adenosine/Adenine Congeners
Source: PLoS One. 2014 May 23;9(5):e97858. doi: 10.1371/journal.pone.0097858 (PMC4032265; doi:10.1371/journal.pone.0097858)

Figure S4. Boundaries of docking boxes. The boundaries of the region explored for docking are highlighted for each studied receptor subtype. The docking grid was built using an inner box (ligand diameter midpoint box, boundaries shown in green) of 10 Å x 10 Å x 10 Å and an outer box (box within which all the ligand atoms must be contained, boundaries shown in purple) that extended 20 Å in each direction from the inner one. The highly conserved Asp 3.32 is shown in spheres in each receptor, as reference point. (A) α_{2B} model (B) α_{2C} model (C) $5HT_{2B}$ crystal (D) $5HT_{2C}$ model (E) $5HT_7$ model and (F) β_3 model.

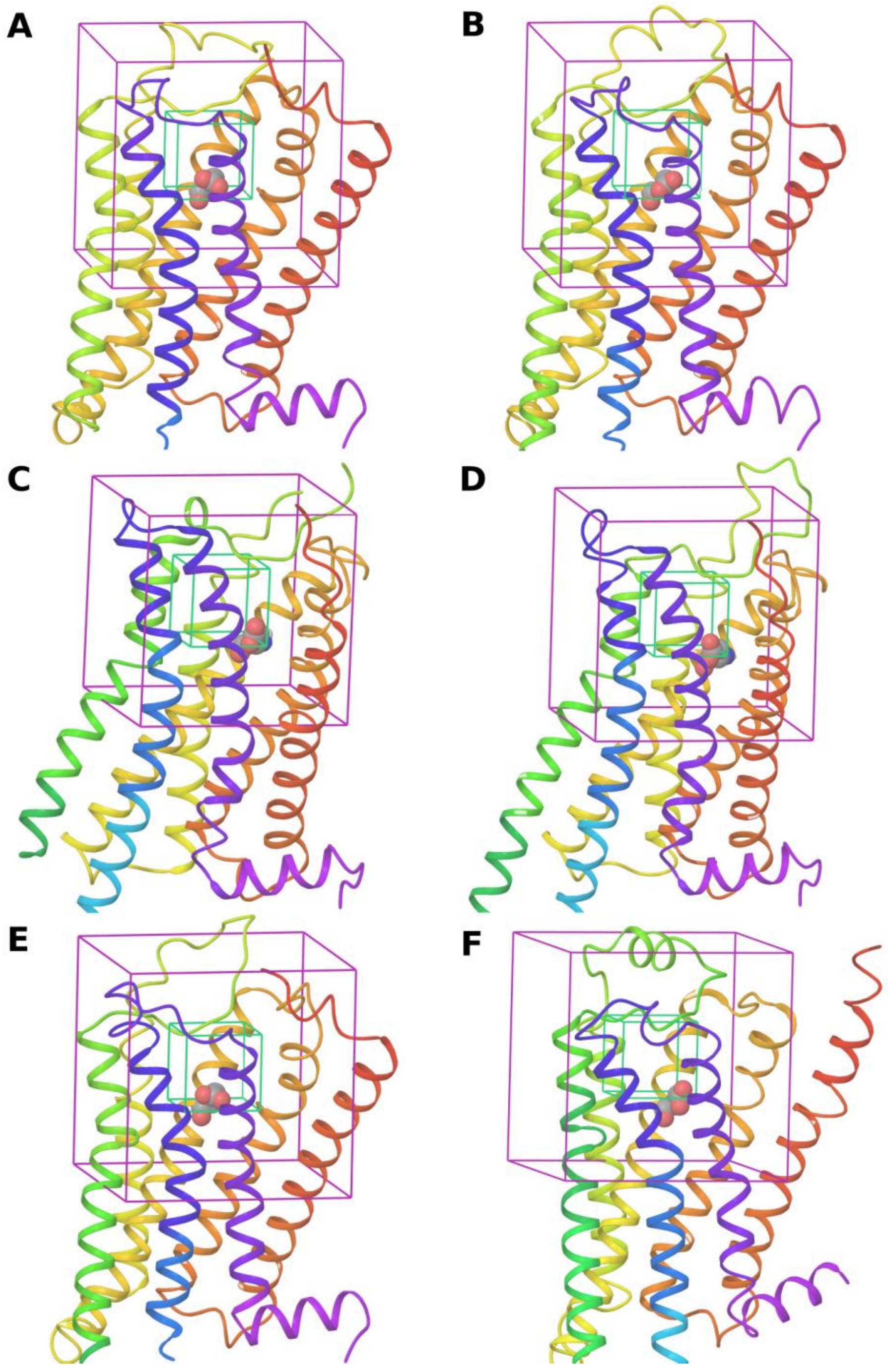

Supplement: Figure S4 — Boundaries of docking boxes. The boundaries of the region explored for docking are highlighted for each studied receptor subtype. The docking grid was built using an inner box (ligand diameter midpoint box, boundaries shown in green) of 10 Å×10 Å×10 Å and an outer box (box within which all the ligand atoms must be contained, boundaries shown in purple) that extended 20 Å in each direction from the inner one. The highly conserved Asp 3.32 is shown in spheres in each receptor, as reference point. (A) α2B model (B) α2C model (C) 5HT2B crystal (D) 5HT2C model (E) 5HT7 model and (F) β3 model. (PDF) [file pone.0097858.s004.pdf]
