# Supplementary material for: Structural Probing of Off-Target G Protein-Coupled Receptor Activities within a Series of Adenosine/Adenine Congeners
Source: PLoS One. 2014 May 23;9(5):e97858. doi: 10.1371/journal.pone.0097858 (PMC4032265; doi:10.1371/journal.pone.0097858)

Figure S5. **Crystallographic poses of ligand complexes as determined by X-ray crystallography and results of self-docking**. Comparison of the crystallographic pose (cyan carbons) and top-scoring docking pose (yellow carbons) of: (A) the biased agonist carvedilol at the turkey β_1 adrenergic receptor (PDB ID: 4AMJ), (B) the agonist ergotamine at the human $5HT_{1B}$ serotonergic receptor (PDB ID: 4IAR) and (C) the agonist ergotamine at the human $5HT_{2B}$ serotonergic receptor (PDB ID: 4IB4). Ligands are shown in ball and stick and some residues important for ligand recognition are shown in stick (gray carbons). Hydrogen atoms are not displayed. H-bonds and salt bridges are shown as black dashed lines. The Connolly surface of the amino acids surrounding the binding site is displayed. Surface color indicates the lipophilic potential: lipophilic regions (green), neutral regions (white) and hydrophilic regions (magenta).

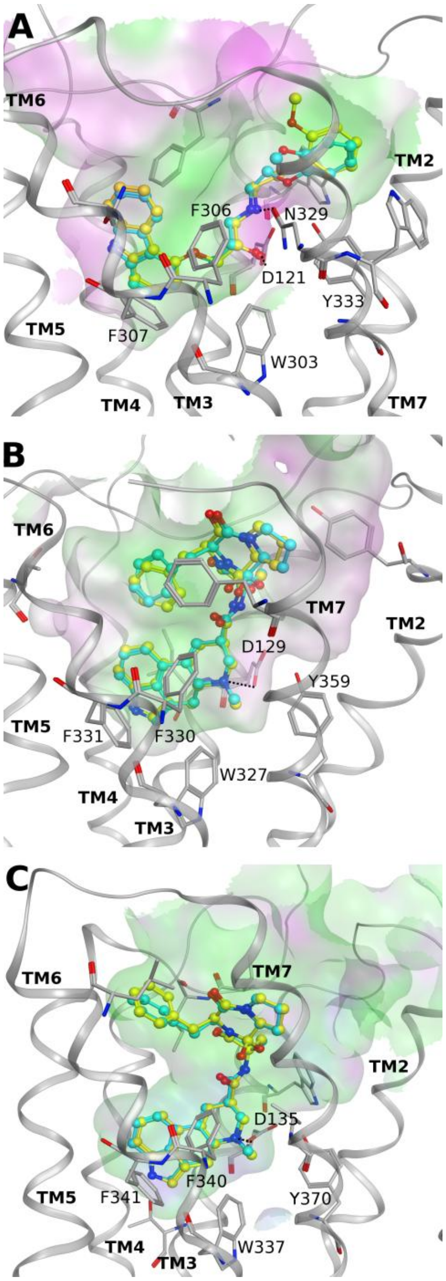

Supplement: Figure S5 — Crystallographic poses of ligand complexes as determined by X-ray crystallography and results of self-docking. Comparison of the crystallographic pose (cyan carbons) and top-scoring docking pose (yellow carbons) of: (A) the biased agonist carvedilol at the turkey β1 adrenergic receptor (PDB ID: 4AMJ), (B) the agonist ergotamine at the human 5HT1B serotonergic receptor (PDB ID: 4IAR) and (C) the agonist ergotamine at the human 5HT2B serotonergic receptor (PDB ID: 4IB4). Ligands are shown in ball and stick and some residues important for ligand recognition are shown in stick (gray carbons). Hydrogen atoms are not displayed. H-bonds and salt bridges are shown as black dashed lines. The Connolly surface of the amino acids surrounding the binding site is displayed. Surface color indicates the lipophilic potential: lipophilic regions (green), neutral regions (white) and hydrophilic regions (magenta). (PDF) [file pone.0097858.s005.pdf]
