# Supplementary material for: Structural Probing of Off-Target G Protein-Coupled Receptor Activities within a Series of Adenosine/Adenine Congeners
Source: PLoS One. 2014 May 23;9(5):e97858. doi: 10.1371/journal.pone.0097858 (PMC4032265; doi:10.1371/journal.pone.0097858)

Figure S6. Docking of known aminergic ligands at target receptors. Results of docking studies performed for known aminergic ligands at selected target receptors (models or crystal structures). Binding modes proposed for: (A) the agonist noradrenaline (cyan carbons) and the antagonist spiroxatrine (magenta carbons) at the human α_{2B} adrenergic receptor model, (B) the antagonists carvedilol (magenta carbons), bupranolol (cyan carbons) and nadolol (yellow carbons) at the human β_3 adrenergic receptor model and (C) the agonist serotonin (cyan carbons) and the antagonist EGIS-7625 (magenta carbons) at the human $5HT_{2B}$ serotonergic receptor (PDB ID: 4IB4). Ligands are shown in ball and stick and some residues important for ligand recognition are shown in stick (gray carbons). Hydrogen atoms are not displayed.

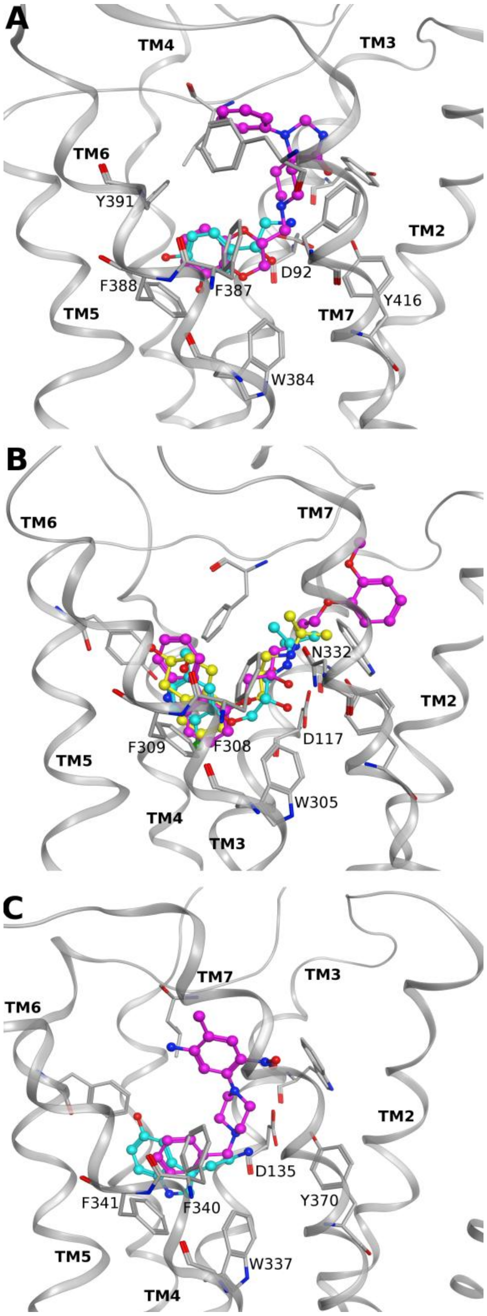

Supplement: Figure S6 — Docking of known aminergic ligands at target receptors. Results of docking studies performed for known aminergic ligands at selected target receptors (models or crystal structures). Binding modes proposed for: (A) the agonist noradrenaline (cyan carbons) and the antagonist spiroxatrine (magenta carbons) at the human α2B adrenergic receptor model, (B) the antagonists carvedilol (magenta carbons), bupranolol (cyan carbons) and nadolol (yellow carbons) at the human β3 adrenergic receptor model and (C) the agonist serotonin (cyan carbons) and the antagonist EGIS-7625 (magenta carbons) at the human 5HT2B serotonergic receptor (PDB ID: 4IB4). Ligands are shown in ball and stick and some residues important for ligand recognition are shown in stick (gray carbons). Hydrogen atoms are not displayed. (PDF) [file pone.0097858.s006.pdf]
