# Supplementary material for: Structural Probing of Off-Target G Protein-Coupled Receptor Activities within a Series of Adenosine/Adenine Congeners
Source: PLoS One. 2014 May 23;9(5):e97858. doi: 10.1371/journal.pone.0097858 (PMC4032265; doi:10.1371/journal.pone.0097858)

Figure S7. Comparison of receptors within the same subfamily. (A) Side view and (B) top view of the superposition of turkey β_1 adrenergic receptor (PDB ID: 4AMJ) (green carbons), human β_2 adrenergic receptor (PDB ID: 2RH1) (cyan carbons) and human β_3 adrenergic receptor model (pink carbons). The second proposed docking pose of compound **3** (orange carbons) as an example at the human β_3 adrenergic receptor model is displayed. For the β_3 adrenergic receptor residues at 4 Å from the ligand are displayed. For the β_1 and β_2 adrenergic receptors only residues at 4 Å from the ligand that differ from the β_3 subtype are displayed. (C) Side view of the superposition of human $5HT_{1B}$ serotonergic receptor (PDB ID: 4IAR) (green carbons), human $5HT_{2B}$ serotonergic receptor (PDB ID: 4IB4) (pink carbons), human $5HT_{2C}$ serotonergic receptor model (orange carbons) and human $5HT_7$ serotonergic receptor model (cyan carbons). (D) top view of the superposition of human $5HT_{1B}$ serotonergic receptor (green carbons) and human $5HT_{2B}$ serotonergic receptor (pink carbons). In C and D the proposed docking pose of compound **1** (yellow carbons) as an example at the human $5HT_{2B}$ serotonergic receptor is displayed. For the $5HT_{2B}$ serotonergic receptor residues at 4 Å from the ligand are displayed. For the $5HT_{1B}$, $5HT_{2C}$ and $5HT_7$ serotonergic receptors only residues at 4 Å from the ligand that differ from the $5HT_{2B}$ subtype are displayed.

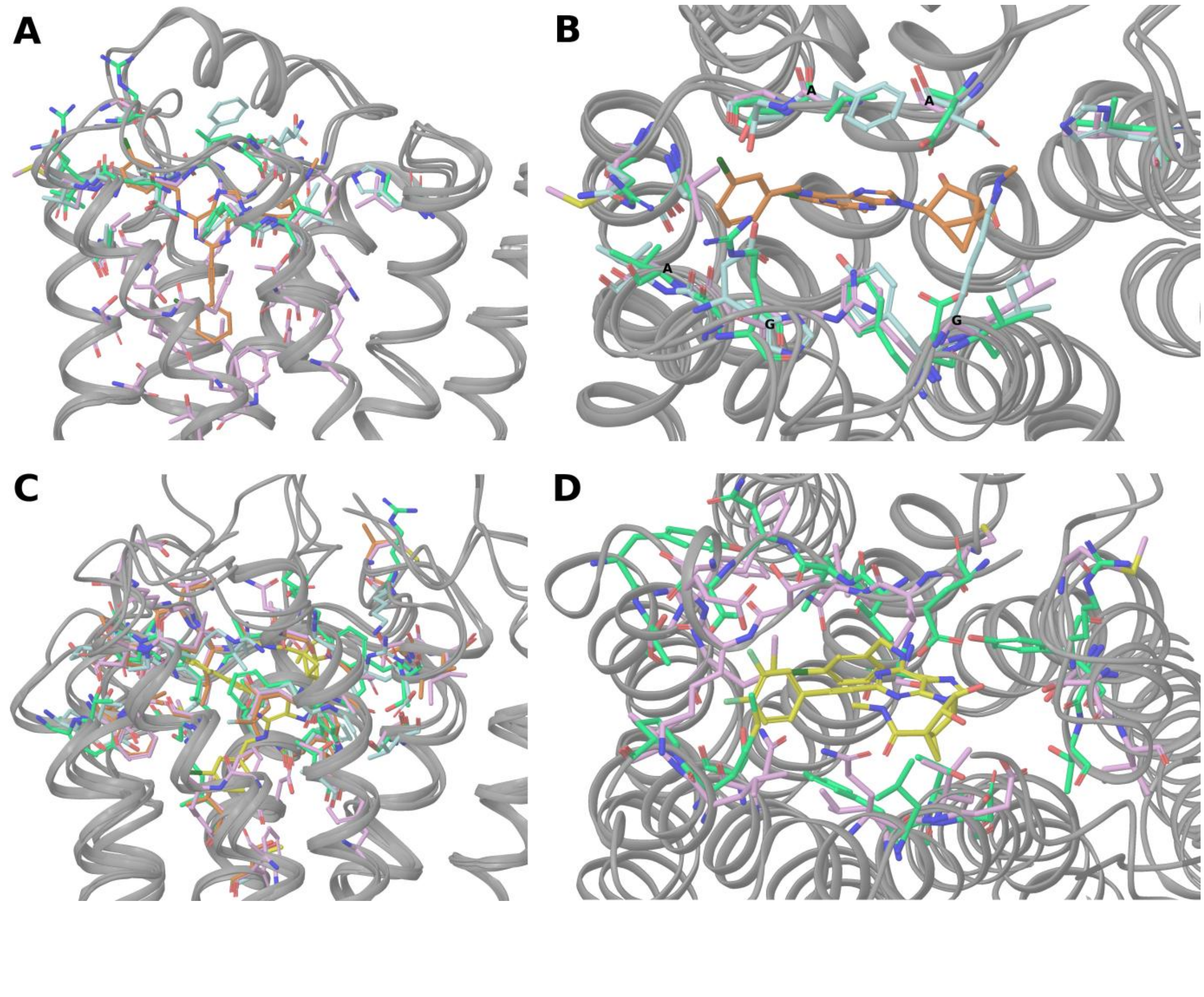

Supplement: Figure S7 — Comparison of receptors within the same subfamily. (A) Side view and (B) top view of the superposition of turkey β1 adrenergic receptor (PDB ID: 4AMJ) (green carbons), human β2 adrenergic receptor (PDB ID: 2RH1) (cyan carbons) and human β3 adrenergic receptor model (pink carbons). The second proposed docking pose of compound 3 (orange carbons) as an example at the human β3 adrenergic receptor model is displayed. For the β3 adrenergic receptor residues at 4 Å from the ligand are displayed. For the β1 and β2 adrenergic receptors only residues at 4 Å from the ligand that differ from the β3 subtype are displayed. (C) Side view of the superposition of human 5HT1B serotonergic receptor (PDB ID: 4IAR) (green carbons), human 5HT2B serotonergic receptor (PDB ID: 4IB4) (pink carbons), human 5HT2C serotonergic receptor model (orange carbons) and human 5HT7 serotonergic receptor model (cyan carbons). (D) top view of the superposition of human 5HT1B serotonergic receptor (green carbons) and human 5HT2B serotonergic receptor (pink carbons). In C and D the proposed docking pose of compound 1 (yellow carbons) as an example at the human 5HT2B serotonergic receptor is displayed. For the 5HT2B serotonergic receptor residues at 4 Å from the ligand are displayed. For the 5HT1B, 5HT2C and 5HT7 serotonergic receptors only residues at 4 Å from the ligand that differ from the 5HT2B subtype are displayed. (PDF) [file pone.0097858.s007.pdf]
