# Supplementary material for: Structural Probing of Off-Target G Protein-Coupled Receptor Activities within a Series of Adenosine/Adenine Congeners
Source: PLoS One. 2014 May 23;9(5):e97858. doi: 10.1371/journal.pone.0097858 (PMC4032265; doi:10.1371/journal.pone.0097858)
Supplement: Table S1 — Binding activity of the adenosine/adenine derivatives 1–10 at three subtypes of human ARs. (PDF) [file pone.0097858.s008.pdf]

Table S1. **Binding activity of the adenosine/adenine derivatives 1-10 at three subtypes of human ARs.**

#	MRS#	hA_1AR *% inhibition* or K_i (nM)	$hA_{2A}AR$ *% inhibition* or K_i (nM)	hA_3AR *% inhibition* or K_i (nM)	Reference
**1**	5698	*(<10%)*	*(41%±10%)*	3.49±1.84	1
**2**	5678	*(20%±6%)*	*(42%±2%)*	2.16±0.34	1
**3**	5697	*(19%±2%)*	*(52%±12%)*	1.92±0.57	1
**4**	3558	260±60	2300±100	0.29±0.04	1
**5**	5676	*(<10%)*	*(<10%)*	1.65±0.08	1
**6**	5755	*(29%±1%)*	1350±190	100±30	2
**7**	5202	1600±150	4520±830	4.9±0.7	4
**8**	5923	*(<10%)*	*(<10%)*	120±17	5
**9**	5930	*(45%±3%)*	*(37%±1%)*	165±34	5
**10**	5474	47.9±10.5	3950±410	470±15	3

1. Tosh, D.K., Deflorian, F., Phan, K., Gao, Z.G., Wan, T.C., Gizewski, E., Auchampach, J.A., Jacobson, K.A. Structure-guided design of A_3 adenosine receptor-selective nucleosides: Combination of 2-arylethynyl and bicyclo[3.1.0]hexane substitutions. J. Med. Chem., 2012, 55:4847-4860.
2. Tosh, D.K., Paoletta, S., Phan, K., Gao, Z.G., Jacobson, K.A. Truncated nucleosides as A_3 adenosine receptor ligands: Combined 2-arylethynyl and bicyclohexane substitutions. ACS Med. Chem. Lett., 2012, 3:596-601.
3. Tosh, D.K., Paoletta, S., Deflorian, F., Phan, K., Moss, S.M., Gao, Z.G., Jiang, X., Jacobson, K.A. Structural sweet spot for A_1 adenosine receptor activation by truncated (N)-methanocarba nucleosides: Receptor docking and potent anticonvulsant activity. J. Med. Chem., 2012, 55:8075–8090.
4. Tosh, D.K., Chinn, M., Ivanov, A.A., Klutz, A. M., Gao, Z.G., Jacobson, K.A. Functionalized congeners of A_3 adenosine receptor-selective nucleosides containing a bicyclo[3.1.0]hexane ring system. J. Med. Chem., 2009, 52:7580-7592.
5. Compounds **8** and **9** are adenine derivatives that were prepared by methods similar to those in references 2 and 4. The compounds were isolated as pure compounds (>98%) and structures confirmed by NMR and high resolution mass spec. Their affinity at ARs was measured for this study, using standard radioligand binding assays as described in references 1-4.
