# Supplementary material for: Structural Probing of Off-Target G Protein-Coupled Receptor Activities within a Series of Adenosine/Adenine Congeners
Source: PLoS One. 2014 May 23;9(5):e97858. doi: 10.1371/journal.pone.0097858 (PMC4032265; doi:10.1371/journal.pone.0097858)
Supplement: Table S2 — Percent inhibition of radioligand binding of the adenosine/adenine derivatives 1–10 in binding to off-target GPCRs, ion channels and a transporter. (PDF) [file pone.0097858.s009.pdf]

Table S2. Percent inhibition of radioligand binding of the adenosine/adenine derivatives 1-10 in binding to off-target GPCRs, ion channels and a transporter

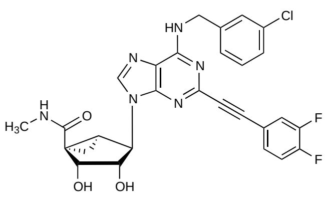

1

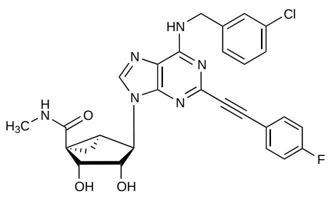

2

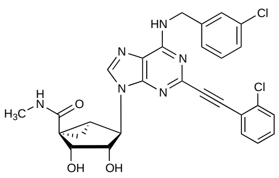

3

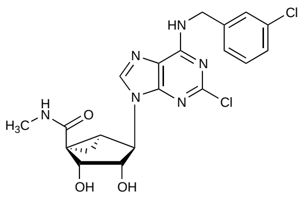

4

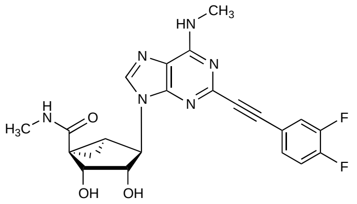

5

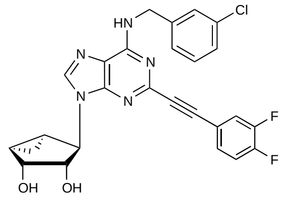

6

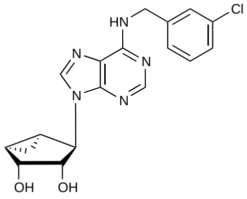

7

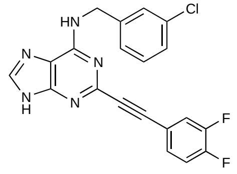

8

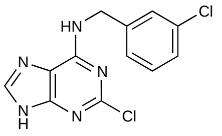

9

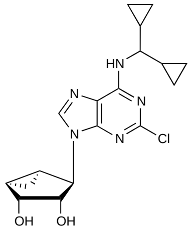

10

		Binding assays<br>% inhibition at 10 μM[a]									
Target	Family	**1**	**2**	**3**	**4**	**5**	**6**	**7**	**8**	**9**	**10**
**GPCRs**											
$\boldsymbol{\alpha_{1A}}$	adrenergic	10	7	16	3	0	13	15	0	26	1
$\boldsymbol{\alpha_{1B}}$	adrenergic	44	1	7	30	11	19	32	30	0	8
$\boldsymbol{\alpha_{1D}}$	adrenergic	3	6	20	0	0	5	30	0	0	13

**α_{2A}**	adrenergic	19[b]	6[b]	22[b]	26[b]	0[b]	14[b]	26[b]	34[b]	58[b]	17[b]
**α_{2B}**	adrenergic	49[b]	12[b]	8[b]	21[b]	0[b]	0[b]	42[b]	63[b]	95[b]	15[b]
**α_{2C}**	adrenergic	63[b]	48[b]	57[b]	11[b]	13[b]	11[b]	51[b]	83[b]	87[b]	0[b]
**β_1**	adrenergic	0[b]	0[b]	2[b]	0[b]	9[b]	6[b]	0[b]	0[b]	37[b]	4[b]
**β_2**	adrenergic	0	0	0	2	2	0	53	0	0	2
**β_3**	adrenergic	74[b]	67[b]	57[b]	0[b]	22[b]	27[b]	4[b]	5[b]	0[b]	0[b]
**M_1**	muscarinic acetylcholine	9[b]	12[b]	0[b]	0[b]	0[b]	3[b]	24[b]	27[b]	12[b]	0[b]
**M_2**	muscarinic acetylcholine	22[b]	0[b]	0[b]	14[b]	0[b]	7[b]	15[b]	6[b]	9[b]	15[b]
**M_3**	muscarinic acetylcholine	2	6	5	1	5	0	11	10	0	0
**M_4**	muscarinic acetylcholine	20	8	8	30	0	32	4	4	0	7
**M_5**	muscarinic acetylcholine	2	9	9	1	4	3	15	2	0	0
**H_1**	histaminergic	0[b]	0[b]	21[b]	6[b]	0[b]	3[b]	64[b]	1[b]	4[b]	0[b]
**H_2**	histaminergic	34	ND	ND	ND	ND	0	ND	ND	ND	13
**H_3**	histaminergic	8	0	0	0	0	0	0	0	3	8
**H_4**	histaminergic	17	28[b]	9[b]	ND	ND	ND	0[b]	5[b]	5[b]	6
**D_1**	dopaminergic	13[b]	19	27	11	0	3	2	0	0	0[b]
**D_2**	dopaminergic	0[b]	0	0	16	18	9	6	0	0	29[b]
**D_3**	dopaminergic	2[b]	4[b]	7[b]	22[b]	4[b]	0[b]	4[b]	23[b]	6[b]	8[b]

**D_4**	dopaminergic	0[b]	0	1	6	4	0	0	0	0	0[b]
**D_5**	dopaminergic	13[b]	19	10	4	6	3	7	9	0	0
**$5HT_{1A}$**	serotonergic	26[b]	5[b]	6[b]	29[b]	10[b]	9[b]	28[b]	11[b]	12[b]	4[b]
**$5HT_{1B}$**	serotonergic	0	0	0	23	15	13	2	32	6	0
**$5HT_{1D}$**	serotonergic	0	21	10	31	13	12	4	26	14	18
**$5HT_{1E}$**	serotonergic	12	0	0	0	9	0	0	0	0	3
**$5HT_{2A}$**	serotonergic	5	19	26	34	11	6	7	9	0	0
**$5HT_{2B}$**	serotonergic	34[b]	56[b]	55[b]	95[b]	4[b]	34[b]	75[b]	39[b]	55[b]	47[b]
**$5HT_{2C}$**	serotonergic	20[b]	16[b]	25[b]	95[b]	1[b]	0[b]	51[b]	11[b]	56[b]	49[b]
**$5HT_{5A}$**	serotonergic	3[b]	25[b]	29[b]	21[b]	5[b]	15[b]	16[b]	11[b]	38[b]	0[b]
**$5HT_6$**	serotonergic	0	36	59	9	15	0	39	0	31	0
**$5HT_7$**	serotonergic	5[b]	6[b]	8[b]	40[b]	0[b]	0[b]	35[b]	0[b]	12[b]	0[b]
**δ**	opioid	44[b]	24[b]	31[b]	10[b]	9[b]	11[b]	7[b]	12[b]	7[b]	0[b]
**κ**	opioid	71	20	19	6	21	29	4	15	0	0
**μ**	opioid	11	7	23	1	5	0	1	15	0	0
**Ion channels**											
**$5HT_3$**	serotonergic	3	26	50	5	2	0	83	0	28	3
**$GABA_A$**	GABA	0	0	0	0	0	ND	0	0	8	11

**Other receptors**											
**σ_1**	Dimethyltryptamine[c]	0	2	33	2	10	16	29	0	0	9
**σ_2**	unknown	0	30[b]	42[b]	0	33	69	66	14	0	0
**Transporter**											
**PBR**	peripheral benzodiazepine	71	92	87	32	25	63	13	13	8	0

[a] All experiments were binding assays performed by the PDSP. % values were from single concentration (10 µM) determination. A value determined as <0% is represented as 0% here (within experimental error). n = 1, unless noted. In some cases K_i values <10 µM are provided in Table 1 for compounds showing low percent inhibition in this table, due to experimental variability of the single point determinations. Single point (10 µM) hERG inhibition was measured in functional assays for compounds: **1**, 43 ± 1.9%; **2**, 5.8 ± 11.9%; **3**, 10.3 ± 4.9%; **10**, 4.5 ± 2.4%.
[b] Mean % inhibition, n = 3.
[c] One of the putative endogenous ligands.

ND, not determined.
