# Supplementary material for: Structural Probing of Off-Target G Protein-Coupled Receptor Activities within a Series of Adenosine/Adenine Congeners
Source: PLoS One. 2014 May 23;9(5):e97858. doi: 10.1371/journal.pone.0097858 (PMC4032265; doi:10.1371/journal.pone.0097858)
Supplement: Table S3 — Physical parameters for each of the adenosine/adenine derivatives 1–10. The total number of off-target interactions (including σ receptors, PBR and one ion channel: 5HT3) determined (in binding assays, unless noted) in this study is indicated. (PDF) [file pone.0097858.s010.pdf]

Table S3. Physical parameters for each of the adenosine/adenine derivatives 1-10. The total number of off-target interactions (including σ receptors, PBR and one ion channel: $5HT_3$) determined (in binding assays, unless noted) in this study is indicated.

#	MW	Rotatable bonds	N atoms	cLogP[a]	H bond donors	tPSA[a]	Number of off-target interactions (K_i <10 μM)
**1**	565	6[b]	6	4.15	4	122	11
**2**	547	6[b]	6	4.08	4	122	6
**3**	563	6[b]	6	4.67	4	122	7[c]
**4**	463	6[b]	6	2.02	4	122	2
**5**	454	4[b]	6	1.99	4	122	2[c]
**6**	508	5	5	4.00	3	92.8	4
**7**	372	4	5	1.13	3	92.8	6
**8**	396	4	5	5.70	2	61.1	5
**9**	294	3	5	3.57	2	61.1	6
**10**	376	5	5	1.41	3	92.8	2

[a] Calculated using ChemBioDraw Ultra (v. 12).

[b] Amide bond is considered nonrotatable. Pseudoglycosidic bond is counted but is likely to remain in a fixed *anti*-conformation.

[c] H_4 antagonist activity from a functional assay.
