# Supplementary material for: Structural Probing of Off-Target G Protein-Coupled Receptor Activities within a Series of Adenosine/Adenine Congeners
Source: PLoS One. 2014 May 23;9(5):e97858. doi: 10.1371/journal.pone.0097858 (PMC4032265; doi:10.1371/journal.pone.0097858)
Supplement: Text S1 — List of all the binding sites for which the primary screening at 10 µM was performed. (PDF) [file pone.0097858.s011.pdf]

Text S1. List of all the binding sites for which the primary screening at 10 μM was performed.

$5HT_{1A}$, $5HT_{1B}$, $5HT_{1D}$, $5HT_{1E}$, $5HT_{2A}$, $5HT_{2B}$, $5HT_{2C}$, $5HT_3$, $5HT_{5A}$, $5HT_6$ and $5HT_7$ serotonergic receptors; α_{1A}, α_{1B}, α_{1D}, α_{2A}, α_{2B}, α_{2C}, β_1, β_2 and β_3 adrenergic receptors; D_1, D_2, D_3, D_4 and D_5 dopaminergic receptors; δ, κ and μ opioid receptors; $GABA_A$ receptor; H_1, H_2, H_3 and H_4 histaminergic receptors; M_1, M_2, M_3, M_4 and M_5 muscarinic receptors; peripheral benzodiazepine receptor (PBR); σ_1 and σ_2 receptors.

Some compounds were additionally assayed at three nicotinic acetylcholine receptors and hERG ion channels. Activity at neurotransmitter transporters is not part of this analysis and will be reported elsewhere. Data were determined by the PDSP. Full procedures are available online at the PDSP web site http://pdsp.med.unc.edu/.
